# FGF13 Deficiency Ameliorates Paclitaxel‐Induced Neuropathic Pain by Inhibiting VASH1‐Mediated Microtubule Detyrosination to Promote Mitophagy

**DOI:** 10.1002/advs.202520995

**Published:** 2026-06-18

**Authors:** Yiming Dong, Yidan Wang, Simeng Lv, Zishan Dong, Kaixi Zhi, Xiuhua Guo, Xuyan Li, Ruoxi Yu, Yiyi Zhang, Siyuan Cheng, Chuan Wang

**Affiliations:** ^1^ Key Laboratory of New Drug Pharmacology and Toxicology Key Laboratory of Neural and Vascular Biology Ministry of Education Hebei Medical University Shijiazhuang China; ^2^ Hebei Key Laboratory of Critical Disease Mechanism and Intervention Department of Pathophysiology Neuroscience Research Center Hebei Medical University Shijiazhuang China; ^3^ College of Basic Medicine Hebei Medical University Shijiazhuang China; ^4^ Affiliated Hospital of Hebei University Clinical Medical College Hebei University Baoding China; ^5^ College of Basic Medical Sciences Hebei University Baoding China; ^6^ Department of Pharmacology Hebei Medical University Shijiazhuang China; ^7^ College of Pharmaceutical Sciences Key Laboratory of Medicinal Chemistry and Molecular Diagnosis College of Life Sciences Ministry of Education Hebei University Baoding China

**Keywords:** fibroblast growth factor 13, microtubules, mitophagy, peripheral neuropathic pain, vasohibin 1

## Abstract

Mitochondrial damage in dorsal root ganglion (DRG) neurons contributes to the pathogenesis of paclitaxel (PTX)‐induced peripheral neuropathic pain (PIPNP). Fibroblast growth factor 13 (FGF13), abundantly expressed in DRG neurons, is crucial for the regulation of somatosensation; however, its role in PIPNP remains unclear. Here, we demonstrated that FGF13 expression is upregulated in DRG neurons of PIPNP model mice. Conditional knockout of *Fgf13* in DRG neurons effectively alleviates PTX‐induced mitochondrial damage and neuropathic pain. RNA sequencing analysis revealed that mitophagy mediates the regulatory effects of FGF13 in PIPNP. Mechanistically, FGF13 physically interacts with vasohibin 1 (VASH1), regulating the binding of VASH1 to microtubules and promoting microtubule detyrosination. FGF13 ablation disrupts assembly of the FGF13‐VASH1‐α‐tubulin ternary complex, impairing VASH1‐mediated microtubule detyrosination and increasing microtubule tyrosination. The resulting accumulation of tyrosinated microtubules facilitates kinesin‐3 (KIF1A)‐driven lysosomal trafficking, which in turn promotes mitophagy activation and ultimately ameliorates PTX‐induced mitochondrial damage and PIPNP. Furthermore, VASH1 overexpression in DRG neurons reversed the alleviating effects of FGF13 deficiency on PTX‐induced mitochondrial damage and PIPNP. In summary, our findings demonstrate that FGF13 deficiency alleviates mitochondrial dysfunction and PIPNP by suppressing VASH1‐dependent microtubule detyrosination and subsequently activating mitophagy. Targeting FGF13 may be a promising therapeutic strategy for PIPNP.

## Graphical abstract

FGF13 is upregulated in DRG neurons of PIPNP model mice. DRG neuron‐specific knockout of FGF13 ameliorates PIPNP symptoms. Mechanistically, FGF13 potentiates microtubule detyrosination by promoting VASH1 binding to microtubules. FGF13 knockout suppresses VASH1‐mediated microtubule detyrosination and promotes α‐tubulin tyrosination. This enhances lysosomal trafficking by KIF1A along tyrosinated microtubules, which activates mitophagy and ultimately ameliorates mitochondrial damage and PIPNP.

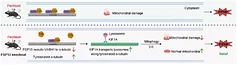

## 1 Introduction

Paclitaxel (PTX) is one of the most commonly utilized chemotherapeutic agents in clinical practice and has been widely applied in the treatment of various solid tumors, including breast cancer, lung cancer, and gastric cancer [1]. PTX‐induced peripheral neuropathic pain (PIPNP) is a common and refractory adverse effect during PTX chemotherapy. It is characterized by persistent numbness, prickling sensations, abnormal temperature sensation, and tactile hyperesthesia in the distal extremities. Typically, these symptoms present in a symmetric “glove and stocking” distribution [2]. Symptoms progressively worsen with each treatment cycle and may persist for months or even years after treatment cessation [3]. PIPNP often seriously impacts the quality of life for individuals surviving cancer, leading to dose reductions or even to cessation of anticancer therapy, which may adversely affect cancer outcomes [4]. Unfortunately, there are currently no drugs recommended for the prevention of PIPNP. Duloxetine remains the only medication recommended for the treatment of PIPNP; however, its efficacy is limited [5]. Therefore, it is imperative to identify effective targets for PIPNP to improve the therapeutic state.

In the nervous system, mitochondria play a critical role in the development and progression of inflammatory and neuropathic pain [6]. Research has demonstrated that PTX induces pain by compromising mitochondrial function in dorsal root ganglion (DRG) neurons [7, 8]. Specifically, PTX reduces superoxide dismutase (SOD) activity while elevating malondialdehyde (MDA) levels via the PI3K/AKT pathway, thereby contributing to mitochondrial damage in DRG neurons [9]. Mitochondria‐targeted antioxidants can suppress PTX‐induced mitochondrial injury and, more significantly, alleviate PTX‐induced mechanical allodynia in rats [10]. Mitophagy is an essential cellular protective mechanism that helps maintain mitochondrial homeostasis by selectively eliminating damaged mitochondria [11]. Damaged mitochondria are enveloped by mitophagosomes and transported along microtubules to lysosomes for fusion and degradation [12]. An intact microtubule network is required for sustaining autophagic flux [13]. In particular, post‐translational modifications (PTMs) of tubulin are crucial in the processes of autophagy [14]. Modifications such as tyrosination and detyrosination influence autophagy by recruiting specific kinesin subtypes, which facilitate the intracellular transport of organelles, including lysosomes. For instance, kinesin‐3 (KIF1A) preferentially binds tyrosinated microtubules to transport cargo vesicles (e.g., lysosomal vesicles) effectively, thereby supporting autophagosome‐lysosome fusion [15, 16, 17]. ‌It has been reported that increasing microtubule acetylation in peripheral nerves can restore axonal transport and mitochondrial function, effectively reversing cisplatin‐induced mechanical allodynia, spontaneous pain, and numbness in mice [18]. Therefore, the identification and study of microtubule regulatory proteins that regulate the PTMs of tubulin are a promising strategy for exploring therapeutic targets for PIPNP and improving clinical outcomes.

Fibroblast growth factor 13 (FGF13) is a member of the non‐secretory fibroblast growth factor family. It is predominantly expressed in the nervous system, where it modulates diverse neuronal populations and contributes to multiple pathophysiological processes [19], including Parkinson's disease [20], inflammatory pain [21], hearing loss [22], and acute and chronic itch [23]. Research has shown that FGF13 binds to microtubules in the central nervous system and regulates their PTMs. As a novel microtubule‐regulatory protein, it influences neuronal polarization and development [24]. Our previous work demonstrated that FGF13 regulates sodium channel function in DRG neurons and inflammatory pain by stabilizing microtubules [21]. Additionally, FGF13 enhances the function of transient receptor potential vanilloid 1 by stabilizing microtubules, thus regulating both acute and chronic pruritus [23]. However, whether FGF13 can regulate PIPNP and the potential underlying mechanisms are largely unknown.

To date, only three tubulin tyrosine carboxypeptidases that catalyze microtubule detyrosination have been identified. These include two heterodimeric complexes, vasohibin 1 (VASH1)/SVBP and vasohibin 2 (VASH2)/SVBP [25, 26], and the monomeric microtubule‐associated tyrosine carboxypeptidase (MATCAP) [27]. Among these, VASH1‐SVBP has been identified as the major tubulin detyrosinase in neurons and cardiomyocytes [25, 28]. Research by our group and others suggests that FGF13 plays a key role in multisystem diseases through its interaction with microtubules, including cardiac diseases [29, 30], platinum‐based drug resistance [31], epilepsy [32], and cancer [33]. However, the specific mechanism by which FGF13 regulates microtubule PTMs has not been investigated.

The present study aimed to determine the role of FGF13 in the pathogenesis of PIPNP and to identify FGF13‐mediated microtubule regulation as a potential therapeutic target for the treatment of PIPNP. To this end, we identified that FGF13, a pivotal regulator of PIPNP, is upregulated in the DRG tissue of PIPNP model mice. By employing sensory neuron‐specific deletion of FGF13, we found that FGF13 deficiency alleviated mitochondrial damage, mechanical allodynia, and thermal hyperalgesia following PIPNP. Mechanistically, knockout of FGF13 reduced the formation of the FGF13‐VASH1‐α‐tubulin ternary complex, thereby inhibiting VASH1‐mediated microtubule detyrosination and increasing microtubule tyrosination levels. This enhances KIF1A‐mediated lysosomal transport along tyrosinated microtubules, which subsequently activates mitophagy, in turn alleviates PTX‐induced mitochondrial damage, and ultimately exerts a protective effect against PIPNP. In summary, this study uncovers a novel mechanism by which FGF13 deletion ameliorates PIPNP through the VASH1‐α‐tubulin axis.

## 2 Results

### 2.1 FGF13 Is Upregulated in DRG Neurons of Mice Challenged With PTX

**FIGURE 1:**
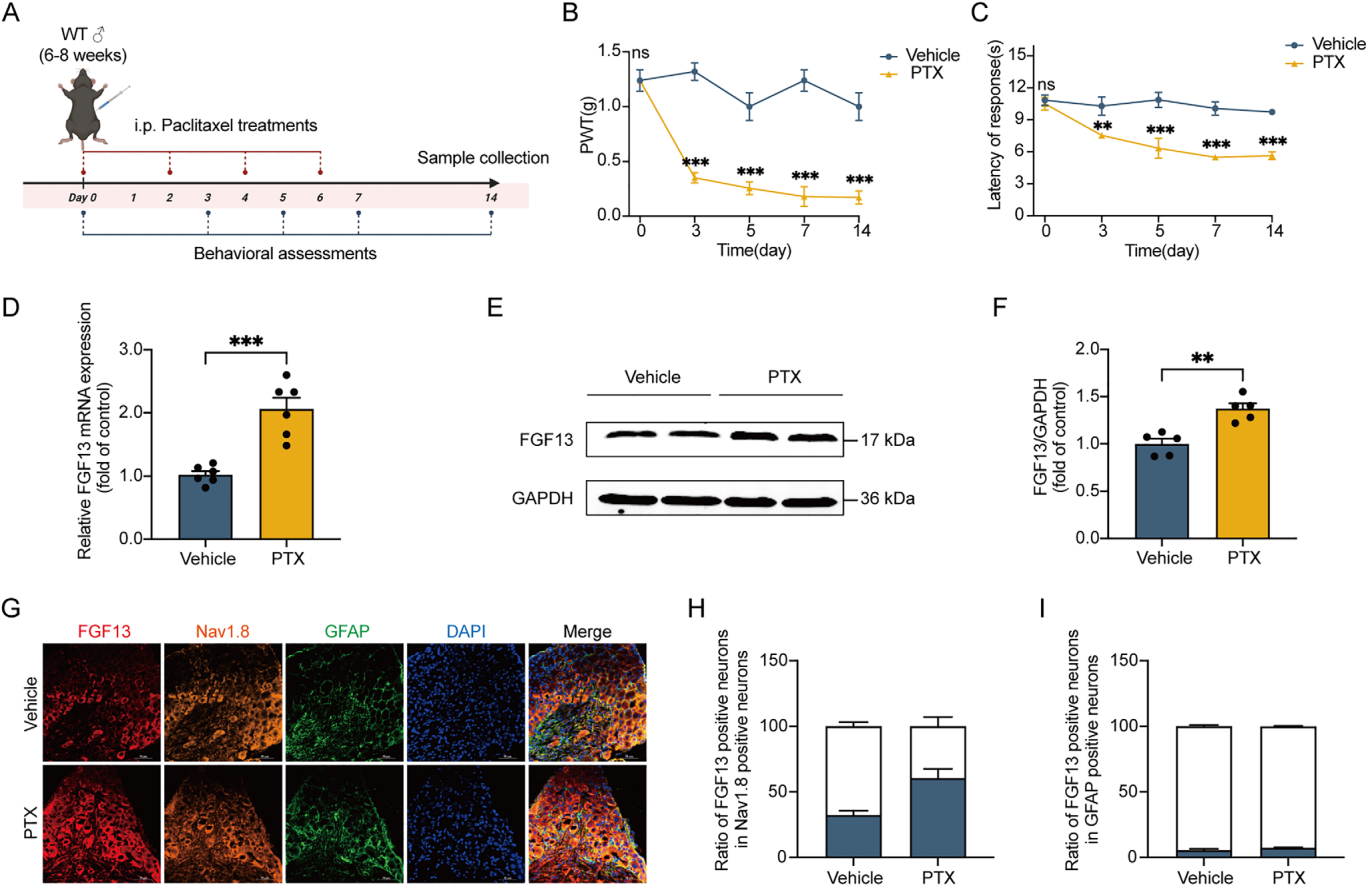
FGF13 is upregulated in DRG neurons of mice challenged with PTX. (A) Schematic diagram of the experimental strategy of PTX‐induced peripheral neuropathic pain in mice (graph created using biorender.com). (B) Mechanical withdrawal threshold and (C) thermal withdrawal latency of WT mice after PTX injection. PWT and PWL were measured one day before and 3, 5, 7, and 14 d after injection (n = 8 per group). (D) mRNA levels of FGF13 following PIPNP were analyzed by RT‐qPCR (normalized to GAPDH) (n = 6 per group). (E,F) Western blotting and quantitative analysis of FGF13 protein levels in DRG tissues (n = 5 per group). (G) Representative immunofluorescence images of FGF13 (red), Nav1.8 (orange), GFAP (green), and DAPI (blue) in mouse DRG tissues. Scale bar: 50 µm. (H) Quantification of the percentage of FGF13‐positive cells within Nav1.8‐positive DRG neurons (n = 3 per group). (I) Quantification of the percentage of FGF13‐positive cells among GFAP‐positive satellite glial cells (n = 3 per group). Data presented as mean ± SEM. ^**^ *p* < 0.01, ^***^ *p* < 0.001. ns, not significant. Two‐tailed unpaired Student's t‐test was used in B, C, E, F, H, I. DRG, dorsal root ganglion; PIPNP, paclitaxel‐induced peripheral neuropathic pain; PTX, paclitaxel; PWT, paw withdrawal mechanical threshold; PWL, paw withdrawal thermal latency; FGF13, fibroblast growth factor 13.

To investigate the relationship between PIPNP and FGF13, we established a PIPNP mouse model via intraperitoneal injection of 8 mg/kg PTX four times every other day to 8‐week‐old male C57BL/6 J mice (Figure 1A). Seven days after the last intraperitoneal PTX injection, mice in the PTX group exhibited significantly reduced mechanical and thermal pain thresholds compared to the control group, confirming successful construction of the PIPNP model (Figure 1B,C). We performed RT‐qPCR analysis and found that FGF13 mRNA levels were increased in DRG neurons of PTX‐treated mice (Figure 1D). Consistently, western blotting data revealed elevated FGF13 levels in DRG neurons from PIPNP mice in comparison with the control group (Figure 1E,F). Furthermore, immunofluorescence analysis of L3‐L5 DRG sections demonstrated that both FGF13 expression levels and the proportion of FGF13‐positive cells were significantly increased in Nav1.8‐positive DRG neurons of PIPNP mice compared to the control group (Figure 1G,H). Notably, FGF13 co‐localized with the neuronal marker Nav1.8 but barely with the satellite glial cell marker GFAP (Figure 1G,I), indicating specific upregulation of FGF13 in Nav1.8‐positive DRG neurons. Therefore, the increased expression of FGF13 may be potentially correlated with PIPNP.

### 2.2 PIPNP Is Attenuated in DRG Neuron‐specific FGF13 Knockout Mice

**FIGURE 2:**
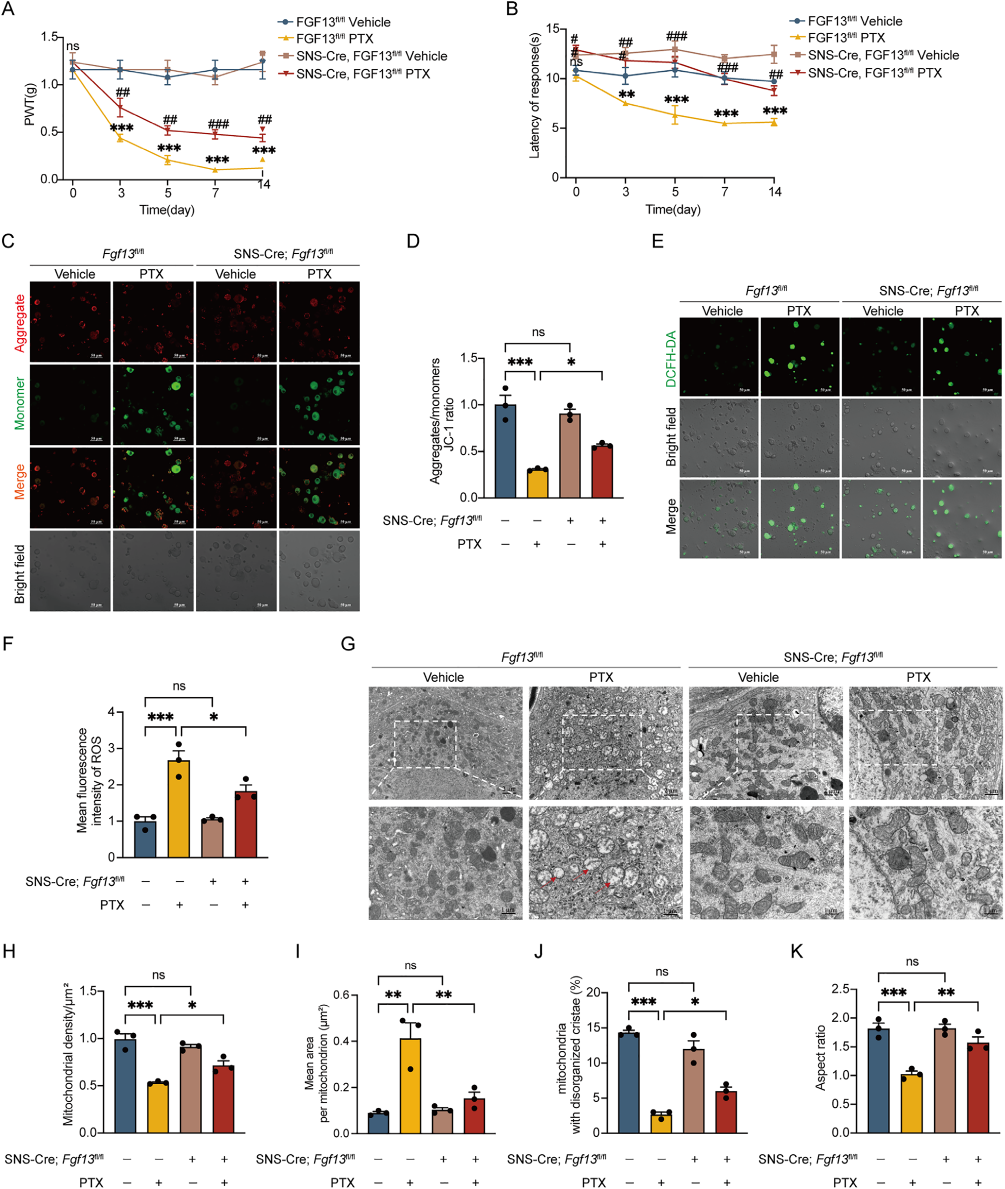
PIPNP is attenuated in DRG neuron‐specific FGF13 knockout mice. (A) Mechanical withdrawal threshold and (B) thermal withdrawal latency of *Fgf13* ^fl/fl^ and SNS‐Cre; *Fgf13* ^fl/fl^ mice after PTX injection (n = 8 per group). (C) The mitochondrial membrane potentials of DRG neurons from *Fgf13* ^fl/fl^ and SNS‐Cre; *Fgf13* ^fl/fl^ mice were determined by JC‐1 staining after treatment with Vehicle or PTX. Scale bar = 50 µm. (D) The ratio of JC‐1 aggregate to JC‐1 monomer was compared in the bar graph (n = 3 per group). (E) Representative confocal images of DCFH‐DA (green) staining in DRG neurons from *Fgf13* ^fl/fl^ and SNS‐Cre; *Fgf13* ^fl/fl^ mice treated with Vehicle or PTX. (F) Bar graph showing relative ROS fluorescence intensity normalized to *Fgf13* ^fl/fl^‐Vehicle group (n = 3 per group). (G) Representative transmission electron microscopy (TEM) images of mitochondrial ultrastructure in DRG tissues from Vehicle‐treated or PTX‐treated *Fgf13* ^fl/fl^ and SNS‐Cre; *Fgf13* ^fl/fl^ mice. Red arrows indicate damaged mitochondria. Scale bars: 2 µm (top), 1 µm (bottom). (H) Mitochondrial density, (I) Mean area per mitochondrion, (J) mitochondria with disorganized cristae, and (K) mitochondrial aspect ratio were quantified from TEM images (n = 3 per group). Data presented as mean ± SEM. ^*^ *p* < 0.05, ^**^ *p* < 0.01, ^***^ *p* < 0.001. ^##^ *p* < 0.01, ^###^ *p* < 0.001. ns, not significant. Two‐way ANOVA followed by Tukey's multiple comparisons test was used in A, B, D, and F. ROS, reactive oxygen species.

To determine the function of FGF13 in PIPNP, we generated DRG neuron‐specific *Fgf13* knockout mice (SNS‐Cre; *Fgf13* ^fl/fl^) by crossing *Fgf13* floxed mice (*Fgf13* ^fl/fl^) with SNS‐Cre mice, which express Cre recombinase under the Nav1.8 promoter (Figure S1A–C). DRG neuron‐specific deletion of FGF13 was confirmed by Western blotting (Figure S1D,E). Hargreaves and Von Frey tests showed comparable mechanical and thermal pain thresholds between *Fgf13* ^fl/fl^ and SNS‐Cre; *Fgf13* ^fl/fl^ mice under physiological conditions (Figure 2A,B). PTX injection significantly reduced mechanical and thermal pain thresholds in *Fgf13* ^fl/fl^ mice, and these hypersensitivities were significantly attenuated in SNS‐Cre; *Fgf13* ^fl/fl^ mice (Figure 2A,B).

Given that mitochondrial damage in DRG neurons plays a pivotal role in PIPNP [7], we next assessed the impact of FGF13 on PTX‐induced mitochondrial damage using JC‐1 staining, reactive oxygen species (ROS) staining, and transmission electron microscopy (TEM). JC‐1 is a mitochondrial potential indicator that exists either as a green fluorescent monomer at depolarized membrane potentials or as a red fluorescent J‐aggregate at hyperpolarized membrane potentials, and the ratio of red to green fluorescence intensity (aggregate/monomer ratio) reflects changes in mitochondrial membrane potential (ΔΨM). The result revealed that PTX treatment increased JC‐1 monomer levels but reduced JC‐1 aggregate formation in DRG neurons, whereas DRG neuron‐specific knockout of FGF13 inhibited this effect (Figure 2C,D). ROS production in DRG tissues was markedly reduced in SNS‐Cre; *Fgf13* ^fl/fl^ mice compared with *Fgf13* ^fl/fl^ mice (Figure 2E,F). We employed TEM to obtain morphological ultrastructure evidence. Consistently, under physiological conditions, FGF13 knockout showed no significant differences compared to controls in mitochondrial number per unit cytoplasmic area, mitochondrial cross‐sectional area, mitochondrial crista density, and mitochondrial aspect ratio, confirming that FGF13 deficiency does not affect baseline mitochondrial homeostasis (Figure 2G–K). In contrast, PTX induced marked mitochondrial damage in *Fgf13* ^fl/fl^ mice, as evidenced by reduced mitochondrial number per unit cytoplasmic area, increased mitochondrial cross‐sectional area, decreased mitochondrial crista density, and decreased mitochondrial aspect ratio. These pathological alterations were significantly ameliorated by FGF13 knockout, as manifested by restored mitochondrial aspect ratio and crista density, normalized mitochondrial cross‐sectional area, and increased mitochondrial number per unit cytoplasmic area (Figure 2G–K).

Taken together, FGF13 plays an important role in maintaining mitochondrial function, and its conditional knockout in DRG neurons alleviates mechanical and thermal pain hypersensitivity in PIPNP.

### 2.3 FGF13 Deletion Induces Mitophagy of DRG Neurons Following PIPNP

**FIGURE 3:**
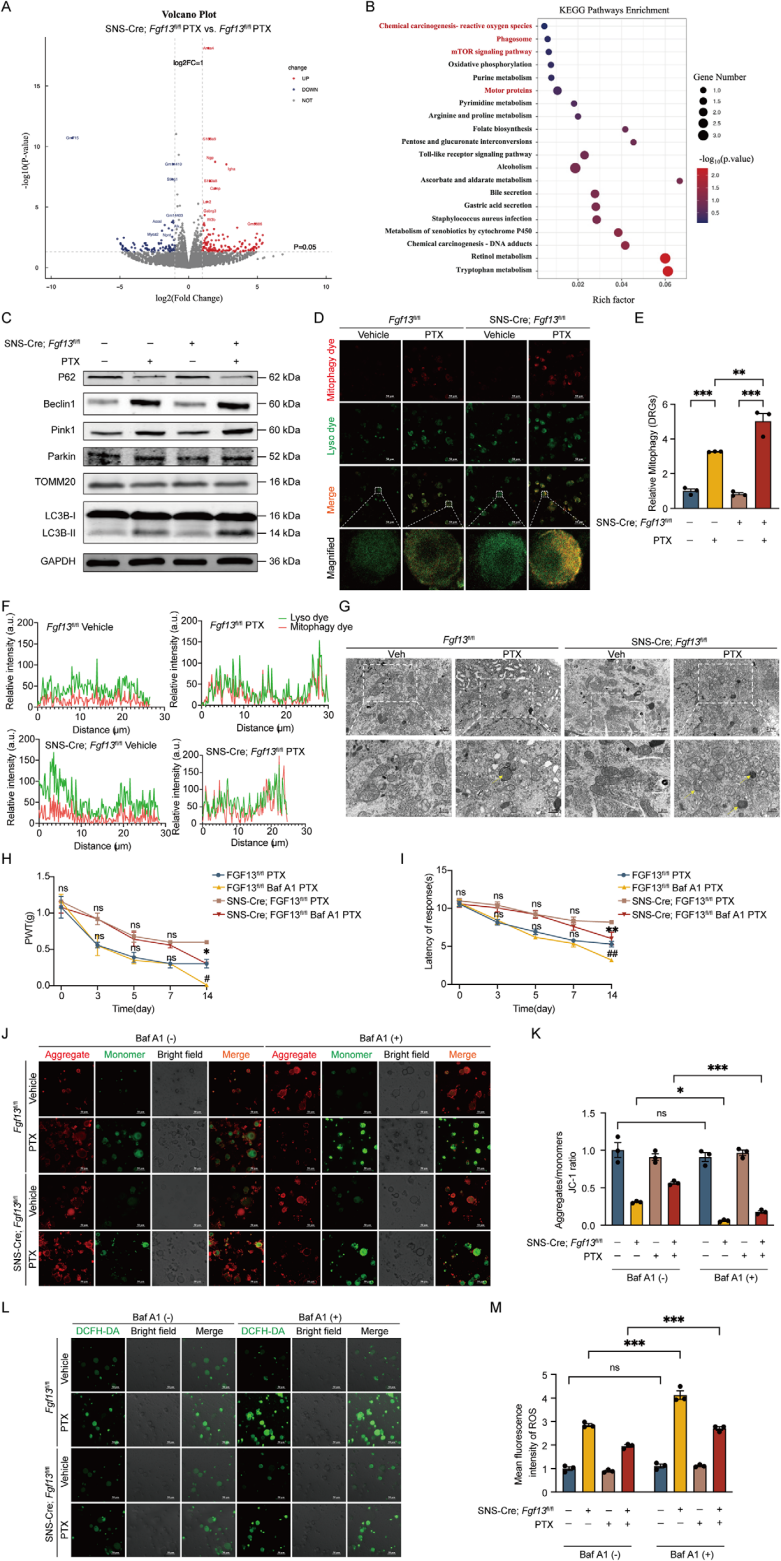
FGF13 deletion induces mitophagy of DRG neurons following PIPNP. (A) Volcano plot of transcriptomics in DRG tissues from *Fgf13* ^fl/fl^ and SNS‐Cre; *Fgf13* ^fl/fl^ mice after PTX induction (red, upregulated genes; blue, downregulated genes; gray, unchanged genes). (B) KEGG pathway enrichment analysis of transcriptomic data from the DRG tissues of *Fgf13* ^fl/fl^ and SNS‐Cre; *Fgf13* ^fl/fl^ mice subjected to PIPNP. (C) Western blot analysis of p62, Beclin1, Pink1, Parkin, TOMM20 and LC3‐II/LC3‐I in DRG tissues from *Fgf13* ^fl/fl^ and SNS‐Cre; *Fgf13* ^fl/fl^ mice treated with Vehicle or PTX (n = 5 per group). (D) Mitophagy and lysosome staining in DRG neurons. Scale bar = 50 µm. (E) The mean fluorescence intensity of mitophagy was quantified (n = 3 per group). (F) Analysis of the colocalization of the mitochondria and lysosome. (G) Representative TEM images of DRG tissues from Vehicle‐treated or PTX‐treated *Fgf13* ^fl/fl^ and SNS‐Cre; *Fgf13* ^fl/fl^ mice. Yellow arrows indicate mitophagy. Scale bars: 2 µm (top), 1 µm (bottom). (H) Mechanical withdrawal thresholds and (I) thermal withdrawal latencies in *Fgf13* ^fl/fl^ and SNS‐Cre; *Fgf13* ^fl/fl^ mice under PIPNP conditions, with or without intrathecal Baf A1 treatment (n = 8 per group). (J) The mitochondrial membrane potentials (ΔΨM) of DRG neurons were determined by JC‐1 staining. Scale bar = 50 µm. (K) The ratio of JC‐1 aggregate to JC‐1 monomer was compared in the bar graph (n = 3 per group). (L) Representative confocal images of DCFH‐DA (green) staining in DRG neurons from *Fgf13* ^fl/fl^ and SNS‐Cre; *Fgf13* ^fl/fl^ mice. M) Quantification of relative ROS fluorescence intensity in DRG neurons (n = 3 per group). Data presented as mean ± SEM. ^*^ *p* < 0.05, ^**^ *p* < 0.01, ^***^ *p* < 0.001. ^#^ *p* < 0.05, ^##^ *p* < 0.01. ns, not significant. Two‐way ANOVA followed by Tukey's multiple comparisons test was used in E, H, I, K, and M. Baf A1, bafilomycin A1; ROS, reactive oxygen species.

To explore the mechanisms by which FGF13 knockout ameliorates PIPNP, we performed comprehensive RNA sequencing (RNA‐seq) analysis on DRG tissues from WT and SNS‐Cre; *Fgf13* ^fl/fl^ mice after PIPNP (Figure S2A). A total of 152 upregulated and 97 downregulated differentially expressed genes (DEGs) were identified using a cutoff of log2‐fold change ≥ 2 with a *p*‐value < 0.05 (Figure 3A). Kyoto Encyclopedia of Genes and Genomes (KEGG) pathway enrichment analysis of these DEGs revealed significant enrichment in mitochondrial damage and autophagy signaling pathways (Figure 3B), suggesting a critical role of FGF13 in regulating mitophagy in DRG neurons.

Mitophagy is a selective autophagic process that specifically eliminates dysfunctional mitochondria, thereby maintaining mitochondrial integrity and cellular homeostasis [34]. To investigate the effects of FGF13 depletion on mitophagy, we examined the protein expression levels of mitophagy markers, including PINK1, Parkin, TOMM20, and Beclin1, as well as autophagy markers LC3‐II/LC3‐I and p62. As shown in Figure 3C and Figure S3A–F, PIPNP induction in *Fgf13* ^fl/fl^ mice resulted in upregulated PINK1 and Parkin expression, increased Beclin1 levels, reduced TOMM20 abundance, and elevated LC3‐II/LC3‐I ratio with concomitant p62 downregulation, and these effects were more pronounced in SNS‐Cre; *Fgf13* ^fl/fl^ mice. Furthermore, to assess the impact of FGF13 knockout on mitophagy, we performed mitophagy and lysosome staining, which allows visualization of mitophagy. Following PIPNP, both the co‐localization of mitochondria and lysosomes and the overlapping signals of Mtphagy Dye and Lyso Dye in DRG neurons were obviously enhanced, and these effects were further intensified by FGF13 deficiency (Figure 3D–F). To gather further evidence supporting the role of FGF13 deficiency in promoting mitophagy in DRG neurons, we utilized TEM to obtain the morphological evidence of mitophagy. Consistently, autolysosome and mitochondrial autophagosome formation were more frequently present in SNS‐Cre; *Fgf13* ^fl/fl^ mice compared to *Fgf13* ^fl/fl^ mice following PIPNP (Figure 3G).

To further explore the role of mitophagy in FGF13‐mediated PIPNP, we administered the autophagy inhibitor bafilomycin A1 (Baf A1) to *Fgf13* ^fl/fl^ and SNS‐Cre; *Fgf13* ^fl/fl^ mice. Hargreaves test and Von Frey test showed mechanical and thermal pain thresholds were not significantly affected by Baf A1 between *Fgf13* ^fl/fl^ and SNS‐Cre; *Fgf13* ^fl/fl^ mice under physiological conditions (Figure S4A,B). Interestingly, in the condition of PIPNP, Baf A1 administration abolished the alleviating effects of FGF13 deletion on PIPNP (Figure 3H,I). JC‐1 staining confirmed that Baf A1 treatment also eliminated the protective effect of FGF13 deletion on mitochondrial damage in DRG neurons (Figure 3J,K). Similar results were observed in ROS staining of DRG neurons (Figure 3L,M). We obtained consistent results using chloroquine (CQ) to block autophagy flux, as CQ treatment similarly reversed the beneficial effects of FGF13 knockout on PIPNP (Figure S5A–D). JC‐1 and ROS staining further demonstrated that CQ blocked the protective effects of FGF13 knockout against PTX‐induced mitochondrial damage, as evidenced by increased JC‐1 monomers, decreased JC‐1 aggregates (Figure S5E,F), and elevated ROS production in DRG neurons (Figure S5G,H). Collectively, these results strongly suggest that FGF13 depletion ameliorates PIPNP by reducing mitochondrial damage through enhanced mitophagy.

### 2.4 FGF13 Modulates PIPNP by Interacting With Microtubule and Promoting Microtubule Tyrosination

**FIGURE 4:**
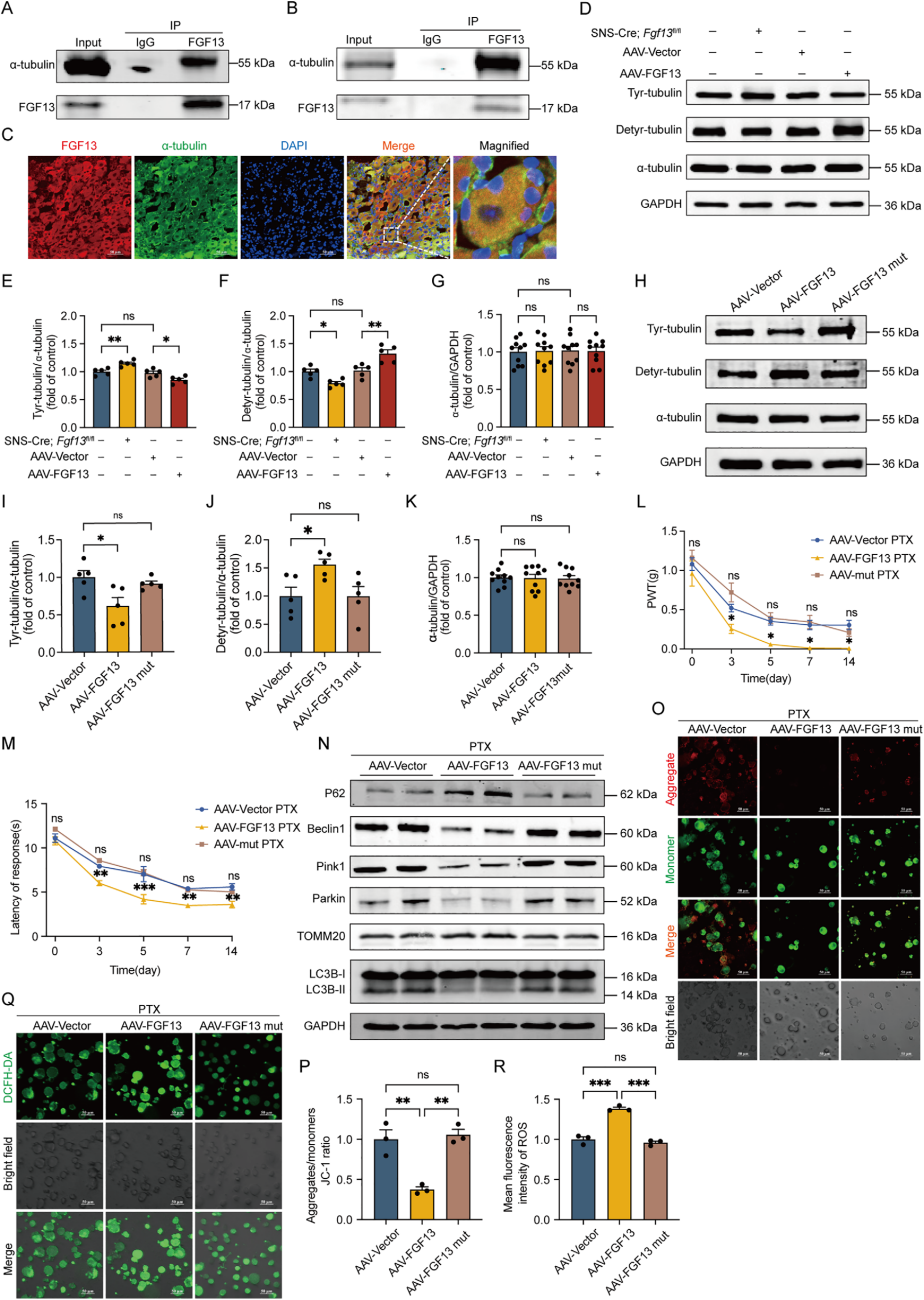
FGF13 modulates mitochondrial damage and PIPNP via microtubule interaction. Interaction between FGF13 and α‐tubulin in DRG neurons was examined by IP‐western blotting assay. IP with FGF13 antibody (A) and IP with α‐tubulin antibody (B). IgG was used as control for IP. (C) Representative immunofluorescence images reveal the colocalization of FGF13 and α‐tubulin in DRG tissues. Nuclei were stained with DAPI (blue). (D) Western blotting analysis revealed the expression levels of Tyr‐tubulin, Detyr‐tubulin, and α‐tubulin in DRG tissues from FGF13 knockout, FGF13 overexpression, and their control mice. (E–G) Quantitative analysis of Tyr‐tubulin, Detyr‐tubulin, and α‐tubulin in the Western blotting experiments (n = 5 per group). (H) Western blotting analysis revealed the expression levels of Tyr‐tubulin, Detyr‐tubulin, and α‐tubulin in DRG tissues from FGF13 overexpression, FGF13 mutant overexpression, and their control mice. (I–K) Quantitative analysis of Tyr‐tubulin, Detyr‐tubulin, and α‐tubulin in the Western blotting experiments (n = 5 per group). (L) Mechanical withdrawal thresholds and (M) thermal withdrawal latencies in FGF13 overexpression, FGF13 mutant overexpression, and their control mice under PIPNP conditions (n = 8 per group). (N) Western blot analysis of p62, Beclin1, Pink1, Parkin, TOMM20 and LC3‐II/LC3‐I in DRG neurons from control mice and mice overexpressing FGF13 or FGF13 mutant under PIPNP conditions (n = 5 per group). (O) JC‐1 staining was used to measure ΔΨM in DRG neurons from control mice and mice overexpressing FGF13 or FGF13 mutant under PIPNP conditions. Scale bar = 50 µm. (P) The ratio of JC‐1 aggregate to JC‐1 monomer was compared in the bar graph (n = 3 per group). (Q) Representative confocal images of DCFH‐DA (green) staining in DRG neurons from control mice and mice overexpressing FGF13 or FGF13 mutant under PIPNP conditions. Scale bar = 50 µm. (R) Quantification of relative ROS fluorescence intensity in DRG neurons (n = 3 per group). Data presented as mean ± SEM. ^*^ *p* < 0.05, ^**^ *p* < 0.01, ^***^ *p* < 0.001. ns, not significant. Two‐way ANOVA followed by Tukey's multiple comparisons test was used in E‐G. One‐way ANOVA followed by Tukey's multiple comparisons test was used in I‐M, P, R. Tyr ‐tubulin, Tyrosinated tubulin; Detyr‐tubulin, Detyrosinated tubulin.

PTMs of tubulin play a critical role in autophagy [14]. FGF13 has been identified as a novel microtubule modifying protein that regulates PTMs of microtubules [24]. We therefore hypothesized that FGF13 influences both mitophagy and PIPNP by modulating the PTMs of tubulin in DRG neurons. In this study, we first verified the physical interaction between FGF13 and α‐tubulin in DRG neurons using co‐immunoprecipitation (Co‐IP) assays (Figure 4A,B). Immunofluorescence co‐localization of FGF13 and α‐tubulin in DRG tissues further showed that both proteins are located in the cytoplasm of DRG neurons, with Pearson's colocalization coefficient analysis confirming their significant spatial correlation (Figure 4C). Next, we examined the levels of detyrosinated microtubules (Detyr‐tubulin) and tyrosinated microtubules (Tyr‐tubulin) in DRG neurons following FGF13 modulation. Western blotting data revealed that FGF13 deletion increased Tyr‐tubulin levels and reduced Detyr‐tubulin levels in DRG neurons (Figure 4D–F). Conversely, overexpression of FGF13 in DRG neurons increased Detyr‐tubulin levels and decreased Tyr‐tubulin levels (Figure 4D–F). Notably, FGF13 did not affect α‐tubulin expression in DRG neurons (Figure 4D,G), indicating that FGF13 functionally targets α‐tubulin.

We next confirmed whether FGF13 regulates mitophagy and PIPNP development through modulating microtubule PTMs in DRG neurons. We constructed a FGF13 mutant (FGF13B^104A‐111A^) viral vector, in which the microtubule‐binding domain was mutated to prevent FGF13 from interacting with microtubules in DRG neurons. Mice were delivered with AAV‐hSyn‐Vector, AAV‐hSyn‐FGF13‐WT and AAV‐hSyn‐FGF13‐Mut 3 weeks prior to PIPNP procedure. AAV‐hSyn‐FGF13‐WT and AAV‐hSyn‐FGF13‐Mut injection significantly elevated FGF13 protein levels in DRG neurons compared with AAV‐hSyn‐Vector (Figure S6A–D). Remarkably, AAV‐hSyn‐FGF13‐WT injection, but not AAV‐hSyn‐FGF13‐Mut, reduced Tyr‐tubulin levels and increased Detyr‐tubulin levels in DRG neurons compared with AAV‐hSyn‐Vector (Figure 4H–K). Behaviorally, mice injected with AAV‐hSyn‐FGF13‐WT displayed heightened mechanical and thermal pain sensitivity in Hargreaves and Von Frey tests (Figure 4L,M). However, AAV‐hSyn‐FGF13‐Mut injection did not reduce the mechanical or thermal pain thresholds in PTX‐treated mice (Figure 4L,M). As shown in Figure 4N and Figure S7A–F, FGF13 overexpression resulted in reduced PINK1 and Parkin expression, decreased Beclin1 levels, increased TOMM20 abundance, elevated p62 expression, and a decreased LC3‐II/LC3‐I ratio, whereas FGF13 mutant overexpression had no effect. JC‐1 staining confirmed that FGF13 overexpression exacerbated mitochondrial damage in DRG neurons of PIPNP mice. In contrast, the degree of mitochondrial damage was not altered upon FGF13 mutant overexpression (Figure 4O,P). Consistently, compared with AAV‐hSyn‐Vector, FGF13 overexpression increased the ROS production in PIPNP mice, whereas no change in ROS production was observed when FGF13 mutant was delivered via AAV the DRG neurons (Figure 4Q,R). Taken together, these data suggest that FGF13 regulates tyrosination/detyrosination of α‐tubulin, and this modulation is an important mechanism by which FGF13 regulates mitophagy, mitochondrial damage, and PIPNP.

**FIGURE 5:**
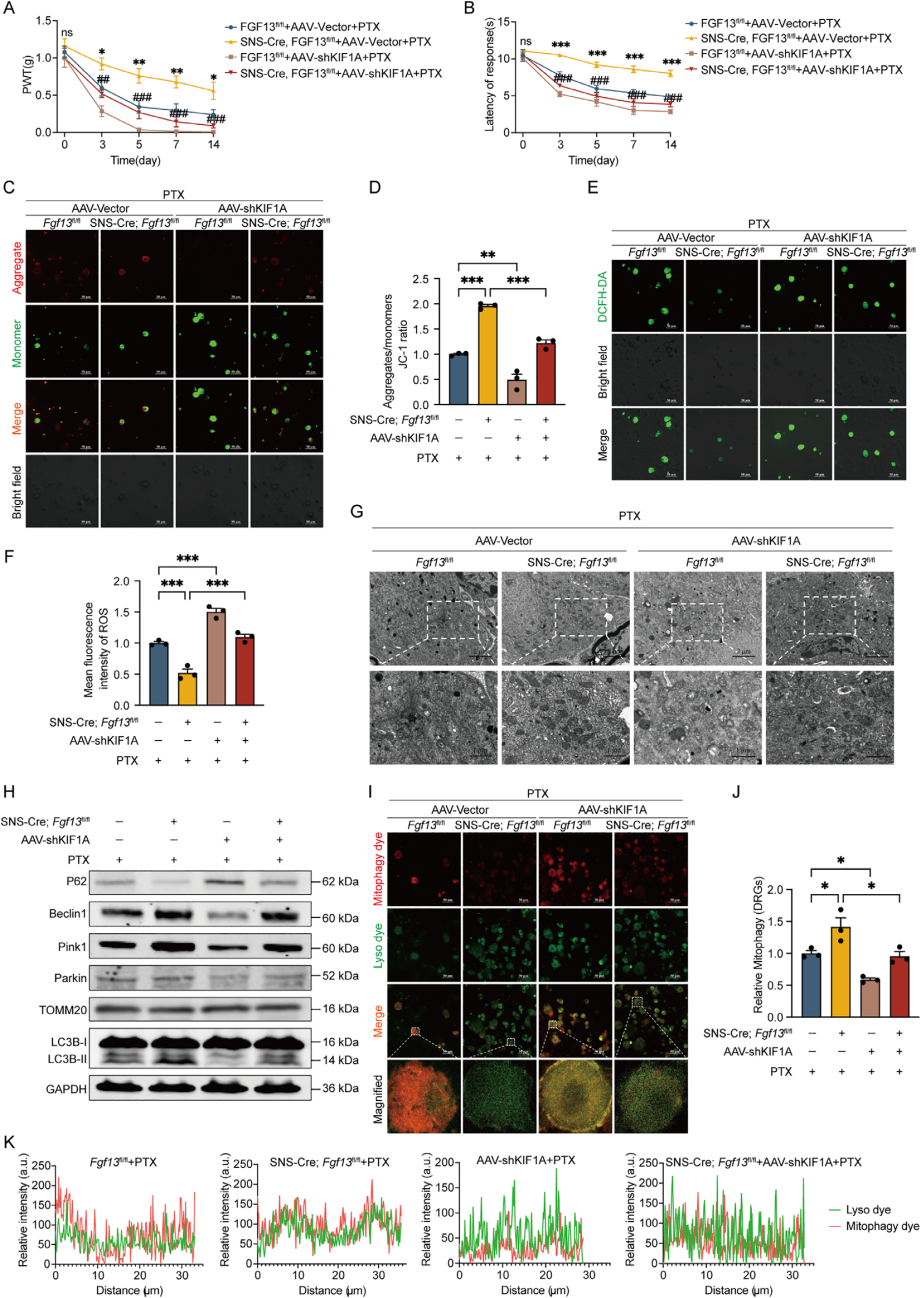
KIF1A is essential for FGF13 deletion‐enhanced mitophagy, attenuation of mitochondrial damage, and alleviation of PIPNP. (A) Mechanical withdrawal thresholds and (B) thermal withdrawal latencies in AAV‐Vector‐ and AAV‐shKIF1A‐treated *Fgf13* ^fl/fl^ and SNS‐Cre; *Fgf13* ^fl/fl^ mice under PIPNP conditions (n = 8 per group). (C) JC‐1 staining was used to measure ΔΨM in DRG neurons from AAV‐Vector‐ and AAV‐shKIF1A‐treated *Fgf13* ^fl/fl^ and SNS‐Cre; *Fgf13* ^fl/fl^ mice under PIPNP conditions. Scale bar = 50 µm. (D) The ratio of JC‐1 aggregate to JC‐1 monomer was compared in the bar graph (n = 3 per group). (E) Representative confocal images of DCFH‐DA (green) staining in DRG neurons from AAV‐Vector‐ and AAV‐shKIF1A‐treated *Fgf13* ^fl/fl^ and SNS‐Cre; *Fgf13* ^fl/fl^ mice under PIPNP conditions. Scale bar = 50 µm. (F) Quantification of relative ROS fluorescence intensity in DRG neurons (n = 3 per group). (G) Representative TEM images of DRG tissues from AAV‐Vector‐ and AAV‐shKIF1A‐treated *Fgf13* ^fl/fl^and SNS‐Cre; *Fgf13* ^fl/fl^ mice under PIPNP conditions. Scale bars: 2 µm (top), 1 µm (bottom). (H) Western blot analysis of p62, Beclin1, Pink1, Parkin, TOMM20 and LC3‐II/LC3‐I in DRG tissues. (I) Mitophagy and lysosome staining in DRG neurons. Scale bar = 50 µm. (J) The mean fluorescence intensity of mitophagy was quantified (n = 3 per group). (K) Analysis of the colocalization of the mitochondria and lysosome. Data presented as mean ± SEM. ^*^ *p* < 0.05, ^**^ *p* < 0.01, ^***^ *p* < 0.001. ^##^ *p* < 0.01, ^###^ *p* < 0.001. ns, not significant. Two‐way ANOVA followed by Tukey's multiple comparisons test was used in A, B, D, F, J.

Since KIF1A transports lysosomes along Tyr‐tubulin to facilitate the interaction and fusion of lysosomes with autophagosomes to initiate autophagy [15], we hypothesized that FGF13 knockout protects against PIPNP‐induced pathology by enhancing Tyr‐tubulin‐mediated mitophagy. To test this, we further explored the role of KIF1A in mediating FGF13‐regulated mitophagy, mitochondrial damage, and PIPNP. To this end, *Fgf13* ^fl/fl^ and SNS‐Cre; *Fgf13* ^fl/fl^ mice were intrathecally injected with AAV‐shKIF1A 4 weeks prior to PIPNP induction. AAV‐shKIF1A injection significantly reduced KIF1A protein levels in DRG neurons compared with AAV‐Vector (Figure S8A,B). Hargreaves test and Von Frey tests revealed that FGF13 knockout significantly alleviated PIPNP‐induced mechanical allodynia and thermal hyperalgesia, which was blocked by KIF1A knockdown (Figure 5A,B). Furthermore, JC‐1 staining and ROS staining showed that FGF13 knockout significantly reduced PIPNP‐induced JC‐1 monomer levels, increased JC‐1 aggregate formation, and reduced ROS production, indicating ameliorated mitochondrial dysfunction; however, all these effects were reversed by KIF1A knockdown (Figure 5C–F). The TEM analyses also demonstrated that FGF13 knockout ameliorated PTX‐induced mitochondrial damage, whereas KIF1A knockdown reversed this protective effect (Figure 5G). Importantly, Western blot and immunofluorescence staining analyses revealed that FGF13 knockout activated mitophagy in DRG neurons, as evidenced by upregulated PINK1 and Parkin expression, increased Beclin1 levels, reduced TOMM20 abundance, elevated LC3‐II/LC3‐I ratio with concomitant p62 downregulation, and enhanced co‐localization of mitochondria with lysosomes, but these responses were prevented by KIF1A knockdown (Figure 5H–K; Figure S9A–F). We then evaluated the role of KIF1A in FGF13‐mediated mitophagy and mitochondrial protection using primary DRG neurons in vitro. In vitro, we utilized DRG neurons from *Fgf13* ^fl/fl^ and SNS‐Cre; *Fgf13* ^fl/fl^ mice and knocked down KIF1A using siRNA (si‐KIF1A). JC‐1 staining and ROS staining showed that mitochondrial damage was obviously reduced in DRG neurons from FGF13‐deficient mice, whereas knockdown of KIF1A abolished this protective effect (Figure S10A–D). In summary, these in vivo and in vitro results demonstrate that FGF13 deficiency alleviates PIPNP by enhancing KIF1A‐mediated lysosomal trafficking along tyrosinated microtubules, thereby promoting mitophagy and alleviating mitochondrial dysfunction.

### 2.5 FGF13 Potentiates Microtubule Detyrosination by Linking VASH1 to Microtubules

Next, we explored how FGF13 regulates microtubule tyrosination. VASH1 piqued our interest as it was identified as a major tubulin detyrosinase in neurons [25]. We therefore hypothesized that FGF13 regulates microtubule detyrosination through VASH1 in DRG neurons. We first verified the physical interaction between VASH1 and α‐tubulin, and explored VASH1's role in regulating α‐tubulin tyrosination. The Co‐IP confirmed the binding between VASH1 and α‐tubulin in DRG neurons (Figure S11A,B). As expected, western blotting data revealed that VASH1 knockdown increased Tyr‐tubulin levels and reduced Detyr‐tubulin levels in DRG neurons (Figure S11C–F).

**FIGURE 6:**
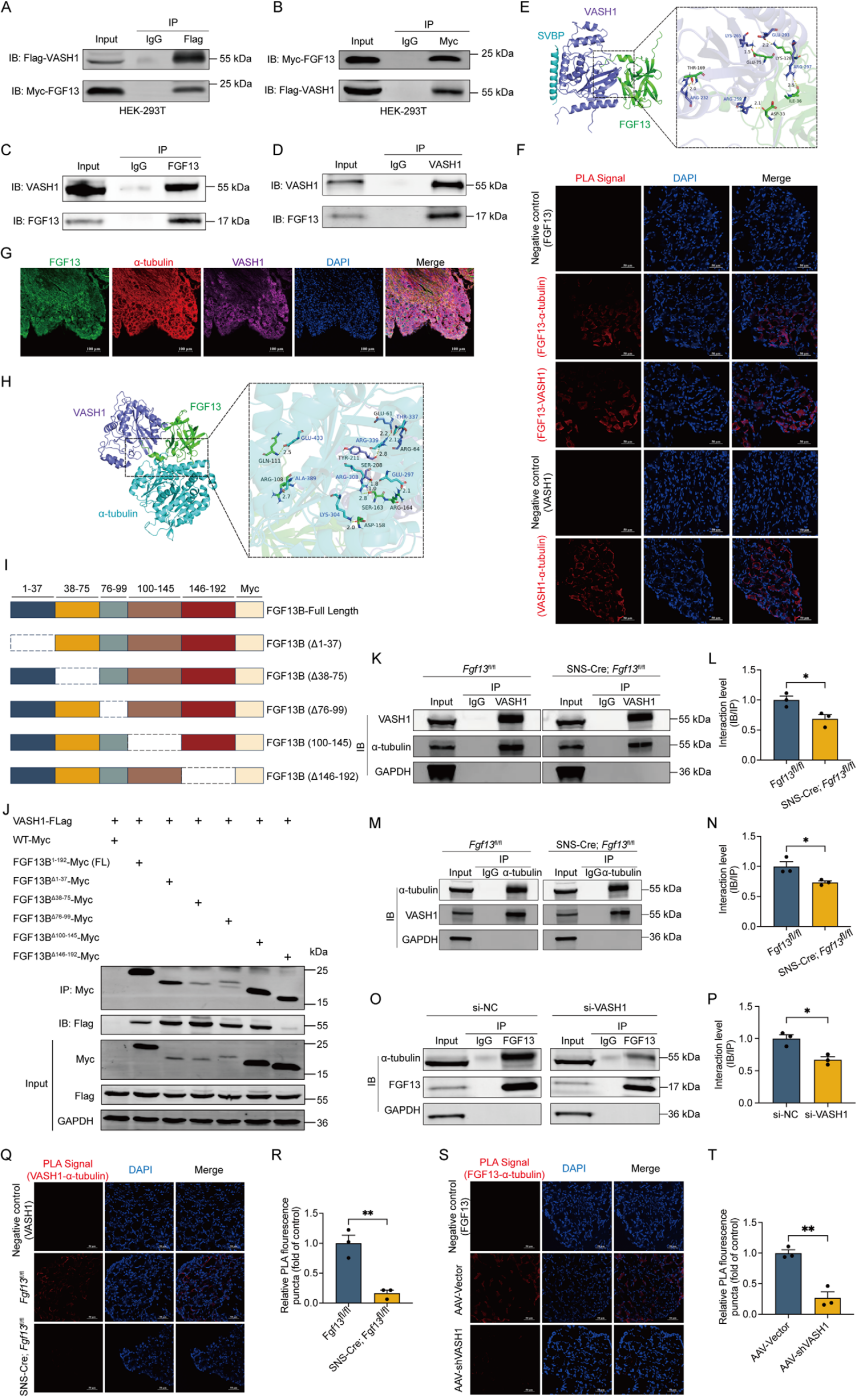
FGF13 potentiates microtubule detyrosination by linking VASH1 to microtubules. (A) HEK‐293T was transfected with Myc‐tagged FGF13 along with Flag‐tagged VASH1. IP analysis with anti‐Flag antibody and immunoblotting with antibodies of anti‐Myc and anti‐Flag, respectively. IgG was used as control for IP. (B) IP analysis with anti‐Myc antibody and immunoblotting with antibodies of anti‐Myc and anti‐Flag, respectively. IgG was used as control for IP. Interaction between FGF13 and VASH1 in DRG tissues was examined by IP‐western blotting assay. IP with FGF13 antibody (C) and IP with VASH1 antibody (D). IgG was used as control for IP. (E) Representative images of molecular docking results to show the potential contact sites between FGF13 (green) and VASH1(purple). (F) Proximity ligation assay (PLA) in DRG tissues demonstrating close interactions between FGF13 and α‐tubulin, FGF13 and VASH1, and VASH1 and α‐tubulin. Scale bars, 50 µm. (G) Representative immunofluorescence images reveal the colocalization of FGF13 (green), α‐tubulin (red) and VASH1 (fuchsia) in DRG tissues. Nuclei were stained with DAPI (blue). (H) Representative images of molecular docking results to show the potential contact sites between FGF13 (green), VASH1(purple), and α‐tubulin (light cyan). (I) Structures of FGF13 full‐length (FL) and truncated plasmids (∆1‐37, ∆38‐75, ∆76‐99, ∆100‐145, ∆146‐192). (J) HEK‐293T cells were co‐transfected with Flag‐tagged VASH1 (VASH1‐Flag) together with either full‐length Myc‐FGF13 (FGF13‐Myc) or its truncated mutants. Interaction between VASH1 and α‐tubulin in DRG tissues of *Fgf13* ^fl/fl^ and SNS‐Cre; *Fgf13* ^fl/fl^ was examined by IP‐western blotting assay. IP with VASH1 antibody (K) and IP with α‐tubulin antibody (M). IgG was used as control for IP. (L) The ratio of IB‐α‐tubulin to IP‐VASH1 was compared in the bar graph (n = 3 per group). (N) The ratio of IB‐VASH1 to IP‐α‐tubulin was compared in the bar graph (n = 3 per group). (O) In the VASH1 knockdown DRG neurons or the control DRG neurons, co‐immunoprecipitation assay was performed to detect the endogenous physical interaction between FGF13 with α‐tubulin. IP analysis with anti‐FGF13 antibody and immunoblotting with antibodies of anti‐FGF13 and anti‐α‐tubulin. (P) The ratio of IB‐α‐tubulin to IP‐FGF13 was compared in the bar graph (n = 3 per group). (Q, R) PLA assay in DRG tissues of *Fgf13* ^fl/fl^ and SNS‐Cre; *Fgf13* ^fl/fl^ mice showed the interaction between VASH1 and α‐tubulin. Scale bar = 50 µm. (S, T) PLA assay in DRG tissues of AAV‐Vector and AAV‐shVASH1 mice showed the interaction between FGF13 and α‐tubulin. Scale bar = 50 µm. Data presented as mean ± SEM. ^*^ *p* < 0.05, ^**^ *p* < 0.01. Two‐tailed unpaired Student's t‐test was used in L, N, P, R, T.

Next, we evaluated the interaction between FGF13 and VASH1 in DRG neurons. HEK‐293T cells were transfected with Myc‐tagged FGF13 along with Flag‐tagged VASH1. Myc‐tagged FGF13 was detected in the Flag‐tagged VASH1 IP complexes following co‐IP assay (Figure 6A). Flag‐tagged VASH1 was also detected in the Myc‐tagged FGF13 IP complexes (Figure 6B). Co‐IP results showed that endogenous FGF13 interacts with VASH1 in DRG neurons. Specifically, VASH1 was detected in anti‐FGF13 immunoprecipitates, and FGF13 was detected in anti‐VASH1 immunoprecipitates, whereas control IgG yielded no such results (Figure 6C,D). We used PyMOL to visualize the predicted results of molecular docking between FGF13 and VASH1. Figure 6E depicts the optimal model of protein docking, with a docking score of ‐242.58‐ a value that increases the likelihood of interaction between FGF13 and VASH1. These results suggest that FGF13 and VASH1 interact in DRG neurons under physiological conditions. To examine the interactions among FGF13, VASH1, and α‐tubulin, we performed a proximity ligation assay (PLA) in DRG tissues. The PLA probe signals indicated close interactions between FGF13 and α‐tubulin, FGF13 and VASH1, as well as VASH1 and α‐tubulin (Figure 6F). Similarly, PLA signals were observed in primary DRG neurons (Figure S12A). Consistently, immunofluorescence showed colocalization between FGF13, VASH1 and α‐tubulin in DRG neurons (Figure 6G). To further validate the formation of the FGF13‐VASH1‐α‐tubulin ternary complex, we performed molecular docking analysis. The docking of the FGF13‐VASH1 protein complex with α‐tubulin yielded a docking score of −204.89 and a confidence score of 0.7498 for the third conformation, indicating high reliability of the predicted ternary complex model (Figure 6H). To decipher the FGF13 sites required for VASH1 interaction, we constructed a series of truncations of the Myc‐tagged FGF13 plasmid and used Co‐IP to clarify the interaction regions (Figure 6I). After co‐transfecting VASH1 and the truncated FGF13 plasmids into HEK‐293T cells, we performed Co‐IP assays. Co‐IP showed that deletion of amino acids 146–192 abolished the interaction of FGF13 with VASH1, suggesting a crucial role for residues 146–192 in the interaction with VASH1 (Figure 6J). Molecular docking analysis further confirmed the interaction between FGF13 residues 146–170 and VASH1 (Figure 6E).

We further elucidated the mechanism by which FGF13 regulates microtubule detyrosination via VASH1. Western blot and RT‐qPCR analyses showed no significant differences in VASH1 protein or mRNA expression levels in DRG tissues between *Fgf13* ^fl/fl^ and SNS‐Cre; *Fgf13* ^fl/fl^ mice (Figure S13A–C). We then performed quantitative Co‐IP using anti‐VASH1 and control IgG antibody in DRG neurons from *Fgf13* ^fl/fl^ and SNS‐Cre; *Fgf13* ^fl/fl^ mice. Remarkably, the interaction between VASH1 and α‐tubulin was markedly diminished in FGF13 knockout DRG neurons (Figure 6K,L). Parallel experiments with anti‐α‐tubulin antibody yielded consistent results (Figure 6M,N). We further determined the interaction between FGF13, VASH1 and α‐tubulin. We performed quantitative Co‐IP to verify the effect of VASH1 on the interaction between FGF13 and α‐tubulin. To this end, we constructed a small interfering RNA to achieve reduced VASH1 expression in DRG neurons. Notably, si‐VASH1 significantly reduced VASH1 protein levels in DRG neurons compared with siNC (Figure S14A,B). Co‐IP showed that VASH1 knockdown led to reduced interactions between FGF13 and α‐tubulin in DRG neurons (Figure 6O,P). To further validate these findings, we performed PLA to visualize the pairwise interactions among FGF13, VASH1, and α‐tubulin. Compared with controls, PLA signals for VASH1‐α‐tubulin interactions were significantly reduced in FGF13 knockout DRG neurons (Figure 6Q,R). Additionally, knockdown of VASH1 in DRG neurons markedly decreased PLA signals for FGF13‐α‐tubulin interactions (Figure 6S,T). We also performed similar experiments in primary DRG neurons and obtained consistent results. PLA signals for VASH1‐α‐tubulin interactions were markedly reduced in FGF13 knockout primary DRG neurons compared with controls (Figure S15A,B). Furthermore, knockdown of VASH1 significantly diminished PLA signals for FGF13‐α‐tubulin interactions (Figure S15C,D). Taken together, these data suggest that FGF13 enhances microtubule detyrosination by bridging VASH1 and α‐tubulin, thereby facilitating their functional interaction.

### 2.6 The VASH1‐α‐Tubulin Axis is Essential for the Attenuation of PIPNP Mediated by FGF13 Deletion

**FIGURE 7:**
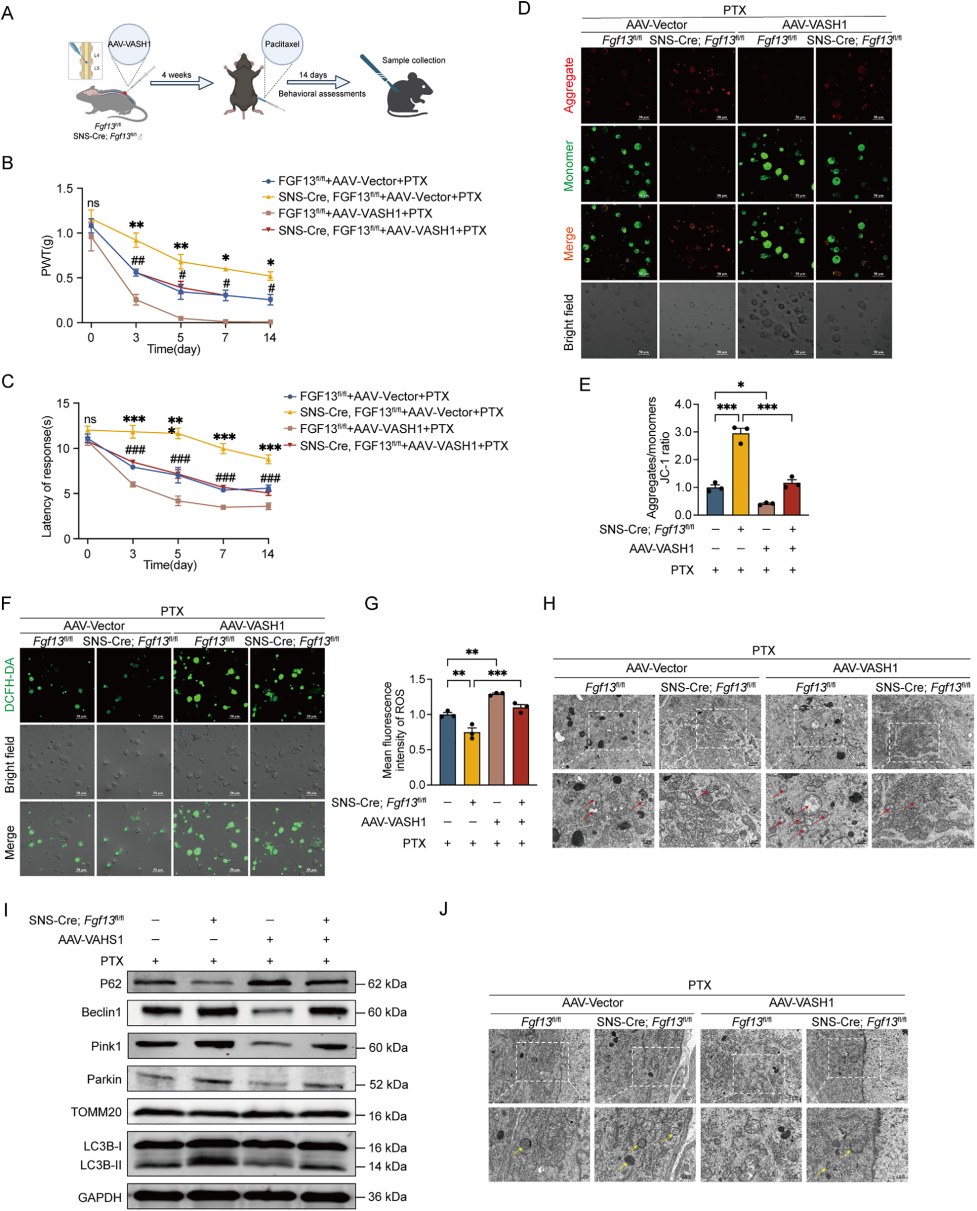
VASH1–α‐tubulin axis is essential for the attenuate effect of FGF13 deletion against PIPNP. (A) Schematic diagram of the experimental strategy for investigating the role of VASH1 in mediating the effect of FGF13 on PIPNP (graph created using biorender.com). (B) Mechanical withdrawal thresholds and (C) thermal withdrawal latencies in AAV‐Vector‐ and AAV‐VASH1‐treated *Fgf13* ^fl/fl^ and SNS‐Cre; *Fgf13* ^fl/fl^ mice under PIPNP conditions (n = 8 per group). (D) JC‐1 staining was used to measure ΔΨM in DRG neurons from AAV‐Vector‐ and AAV‐VASH1‐treated *Fgf13* ^fl/fl^ and SNS‐Cre; *Fgf13* ^fl/fl^ mice under PIPNP conditions. Scale bar = 50 µm. (E) The ratio of JC‐1 aggregate to JC‐1 monomer was compared in the bar graph (n = 3 per group). (F) Representative confocal images of DCFH‐DA (green) staining in DRG neurons from AAV‐Vector‐ and AAV‐VASH1‐treated *Fgf13* ^fl/fl^ and SNS‐Cre; *Fgf13* ^fl/fl^ mice under PIPNP conditions. Scale bar = 50 µm. (G) Quantification of relative ROS fluorescence intensity in DRG neurons (n = 3 per group). (H) Representative TEM images of DRG tissues from AAV‐Vector‐ and AAV‐VASH1‐treated *Fgf13* ^fl/fl^and SNS‐Cre; *Fgf13* ^fl/fl^ mice under PIPNP conditions. Red arrows indicate damaged mitochondria. Scale bars: 2 µm (top), 1 µm (bottom). (I) Western blotting of p62 and LC3‐II/LC3‐I in DRG tissues. (J, K) Quantitative analysis of p62 and LC3‐II/LC3‐I (n = 5 per group). The protein level was standardized by GAPDH. (L) Representative TEM images of DRG tissues from AAV‐Vector‐ and AAV‐VASH1‐treated *Fgf13* ^fl/fl^ and SNS‐Cre; *Fgf13* ^fl/fl^ mice under PIPNP conditions. Yellow arrows indicate mitophagy. Scale bars: 2 µm (top), 1 µm (bottom). Data presented as mean ± SEM. ^*^ *p* < 0.05, ^**^ *p* < 0.01, ^***^ *p* < 0.001. ^#^ *p* < 0.05, ^##^ *p* < 0.01, ^###^ *p* < 0.001. ns, not significant. Two‐tailed unpaired Student's t‐test was used in B, C, E, G.

We further explored whether FGF13 deletion‐evoked PIPNP attenuation is dependent upon VASH1. To this end, *Fgf13* ^fl/fl^ and SNS‐Cre; *Fgf13* ^fl/fl^ mice were intrathecally injected with AAV‐hSyn‐VASH1 4 weeks prior to PIPNP induction. A brief flow chart of this experiment is shown in Figure 7A. AAV‐hSyn‐VASH1 injection significantly elevated VASH1 protein levels in DRG neurons compared with AAV‐hSyn‐Vector (Figure S16A,B). Notably, FGF13 deletion reduced Detyr‐tubulin levels and increased Tyr‐tubulin levels in DRG neurons, which were reversed by DRG neuron‐specific VASH1 overexpression (Figure S16C–F). Hargreaves test and Von Frey test showed that DRG neuron‐specific FGF13 knockout in mice significantly attenuated PIPNP‐induced mechanical and thermal pain, which was blocked by VASH1 overexpression (Figure 7B,C). Furthermore, FGF13 knockout significantly reduced PIPNP‐evoked JC‐1 monomer levels, increased JC‐1 aggregate formation, and reduced ROS production, all of which were again reversed by overexpression of VASH1 (Figure 7D–G). The TEM analyses showed that FGF13 knockout ameliorates PTX‐induced mitochondrial damage, but VASH1 overexpression reversed this protective effect (Figure 7H). Furthermore, Western blot analysis revealed that FGF13 knockout activated mitophagy in DRG tissues during PIPNP, as evidenced by upregulated PINK1 and Parkin expression, increased Beclin1 levels, reduced TOMM20 abundance, elevated LC3‐II/LC3‐I ratio with concomitant p62 downregulation, but these responses were prevented by VASH1 overexpression (Figure 7I; Figure S17A–F). Lastly, TEM analyses showed that FGF13 knockout in DRG neurons activated mitophagy, but this response was prevented by AAV‐hSyn‐VASH1 injection in mice (Figure 7J). Taken together, these results demonstrate that FGF13 knockout ameliorates PTX‐induced mitochondrial damage and PIPNP by activating mitophagy in a manner dependent on the VASH1/α‐tubulin axis.

## 3 Discussion

**FIGURE 8:**
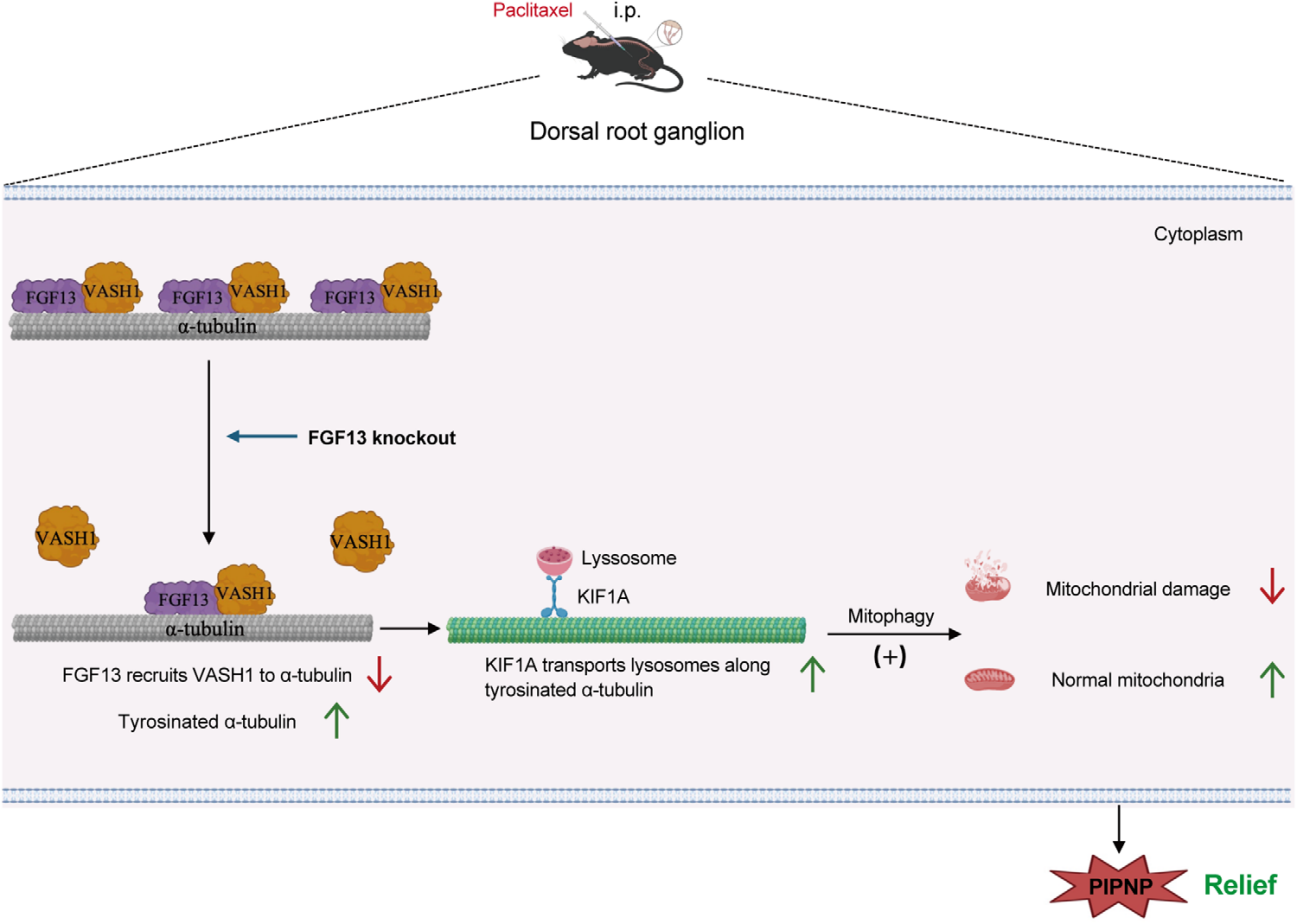
Schematic diagram of the mechanism of FGF13 downregulation to alleviate PIPNP. FGF13 deficiency disrupts the FGF13‐VASH1‐α‐tubulin ternary complex, thereby promoting microtubule tyrosination and subsequent KIF1A‐mediated lysosomal trafficking. This enhanced mitophagy ameliorates PTX‐induced mitochondrial dysfunction and protects against PIPNP.

In this study, we demonstrated a crucial role for FGF13 in mediating PIPNP. FGF13 expression was upregulated in PTX‐challenged mouse DRG neurons. PIPNP is attenuated in DRG neuron‐specific FGF13 knockout mice. Mechanistically, FGF13 depletion ameliorates PIPNP by regulating mitophagy to reduce mitochondrial damage. Our further analyses revealed that FGF13 modulates mitochondrial damage in PIPNP by interacting with microtubules and promoting microtubule detyrosination. Furthermore, we provide the first evidence that FGF13 acts as a scaffold protein and promotes the interaction between VASH1 and microtubules by forming a ternary protein complex with them, thereby enhancing the levels of microtubule detyrosination. FGF13 depletion ameliorates PTX‐induced mitochondrial damage and PIPNP through activation of mitophagy in a VASH1/α‐tubulin‐dependent manner. (graphic abstract shown in Figure 8). These findings suggest that FGF13 is a potential therapeutic target for PIPNP and uncover the distinct mechanism by which FGF13 regulates tyrosination of microtubules.

PTX is a well‐established antitumor agent, yet it concurrently induces neurotoxic side effects, predominantly inducing PIPNP that can persist long after drug withdrawal [2, 35]. PIPNP significantly impairs clinical outcomes and reduces the quality of life in cancer patients [4]. Currently, PIPNP treatment remains suboptimal, largely due to its complex pathogenesis and the limited number of known therapeutic targets [5, 8]. The quest for therapeutic targets underlying the pathogenesis of PIPNP is critical for devising specific intervention strategies. As the primary sensory neurons for nociceptive afferents, DRG neurons play a crucial role in pain transmission [36]. We observed upregulated FGF13 expression in DRG neurons of PIPNP mice. Conditional knockout of FGF13 in DRG neurons effectively alleviated PTX‐induced mechanical allodynia and thermal hyperalgesia in mice. These findings suggest that FGF13 could be a promising target for modulating PIPNP.

We next explored how FGF13 regulates PIPNP. RNA sequencing revealed that FGF13 depletion alters genes involved in mitochondrial damage and autophagy pathways. Mitochondria play a critical role in the onset and progression of both inflammatory and neuropathic pain, and mitochondrial damage can directly trigger nociceptive responses [37]. Mitochondrial damage also plays a pivotal role in the pathogenesis of PIPNP [7]. Our data showed that FGF13 deletion ameliorated PTX‐induced mitochondrial damage. Mitophagy, a selective autophagy process, eliminates dysfunctional mitochondria to maintain cellular homeostasis [38]. We also found that FGF13 deficiency significantly enhanced mitophagy, as revealed by the elevated expression of autophagy markers and the increased co‐localization of mitochondria and lysosomes, together with markedly enhanced overlapping signals of Mtphagy Dye and Lyso Dye in DRG tissue from SNS‐Cre; *Fgf13* ^fl/fl^ mice. Next, we performed mitophagy‐rescue experiments to clarify the relationship between FGF13 and mitophagy in the regulation of PIPNP. Therefore, we conducted in vivo experiments in which Baf A1 and CQ were used to pharmacologically inhibit mitophagy, thereby verifying the dependency of FGF13‐mediated modulation of PIPNP on mitophagy. Pharmacological inhibition of mitophagy abolished the protective effects of FGF13 knockout on PTX‐induced mitochondrial dysfunction and PIPNP. Therefore, these findings revealed that FGF13 knockout ameliorates PTX‐induced mitochondrial damage and neuropathic pain through the activation of mitophagy.

We then explored how FGF13 regulates mitophagy in DRG neurons within PIPNP models. Microtubules participate in multiple stages of autophagy, serving as global and local integrators of the autophagy‐lysosome pathway. In collaboration with the ubiquitin‐proteasome system, they are essential for maintaining proteostasis in cardiomyocytes [13, 14]. Notably, microtubule PTMs play a critical role in the autophagic process [14]. FGF13 binds to tubulin and regulates microtubule stability through modulating these microtubule PTMs [24]. Our previous studies demonstrated that FGF13 significantly influences inflammatory pain [21], itch sensation [23], and heart failure [29] by regulating microtubule stability. We therefore sought to determine whether FGF13 regulates mitophagy and PIPNP by modulating microtubule PTMs. Consistent with previous reports [21, 39], FGF13 deficiency significantly increased Tyr‐tubulin levels and reduced Detyr‐tubulin levels in DRG neurons. Conversely, overexpression of FGF13 increased Detyr‐tubulin levels and reduced Tyr‐tubulin levels. To investigate whether the regulation of mitochondrial damage and PIPNP by FGF13 is mediated by microtubules, we generated FGF13 mutants designed to disrupt its binding to microtubules. We found that overexpression of FGF13 in DRG neurons exacerbated PTX‐induced mitochondrial damage and neuropathic pain. Importantly, overexpression of the FGF13 mutant had no significant effect on either mitochondrial damage or the severity of PIPNP. These findings suggest that FGF13‐mediated tyrosination of microtubules represents the pivotal mechanism underlying FGF13's regulation of PIPNP.

Microtubules serve as tracks for kinesin‐based intracellular transport and are essential for maintaining cellular architecture and intracellular trafficking [40]. Kinesins are essential molecular motors that transport cellular cargo along microtubules [41]. Kinesins exhibit distinct motility characteristics on microtubules with different PTMs. Some kinesin superfamily members preferentially interact with specific subsets of microtubules that are identified by their PTM status [40, 42]. For example, kinesin‐3 family members, specifically KIF1A and KIF1Bβ, preferentially bind to tyrosinated microtubules [15]. KIF1A mediates lysosomal trafficking along tyrosinated microtubules [15]. We demonstrated that increased microtubule tyrosination ameliorates PTX‐induced mitochondrial dysfunction and neuropathic pain. To test whether KIF1A mediates this effect, we performed KIF1A knockdown experiments, which showed that KIF1A knockdown reversed the protective effects of FGF13 deletion against mitochondrial damage and PIPNP. These findings indicate that FGF13 regulates mitophagy, mitochondrial damage, and PIPNP through KIF1A.

Given the critical role of FGF13 in the heart [29, 30], nervous system [32], and cancer [33] by regulating microtubule PTMs, we further explored the downstream signalling targets of FGF13, focusing specifically on VASH1. VASH1 was identified as a major tubulin detyrosinase in neurons and cardiomyocytes [25]. The enzymatic complex formed by the interaction of VASH1 and SVBP binds to microtubules, thereby regulating microtubules’ detyrosination. This modulation influences the properties of neuronal axons and is crucial for various neuronal physiological processes [43]. As FGF13 lacks catalytic domains, we hypothesized it regulates microtubule detyrosination via VASH1 in DRG neurons. Our findings indicate that VASH1 binds to microtubules and decreases microtubule tyrosination while increasing microtubule detyrosination, which is in agreement with previous observations [25]. In our study, Co‐IP and protein‐protein docking analyses demonstrated an interaction between FGF13 and VASH1. We further mapped the VASH1‐binding domain on FGF13 and demonstrated that FGF13 interacts physically with VASH1 through amino acid residues 146–192. We found that FGF13 deletion markedly reduced VASH1‐α‐tubulin binding in DRG neurons. Additionally, Co‐IP and PLA analyses confirmed that FGF13's binding to α‐tubulin decreases upon VASH1 knockdown. These data establish FGF13 as a scaffold protein that nucleates a ternary complex with VASH1 and α‐tubulin to coordinate microtubule tyrosination. While our data demonstrate that FGF13 bridges VASH1 and α‐tubulin to facilitate microtubule detyrosination, the current experiments cannot definitively distinguish whether FGF13 primarily functions as a scaffold to enhance VASH1‐α‐tubulin proximity or directly modulates VASH1 enzymatic activity. This limitation arises from the inherent nature of the VASH1‐SVBP complex, where α‐tubulin serves as both the obligate substrate and the essential binding partner for catalytic activity. Future studies to determine whether FGF13 directly influences VASH1 catalytic kinetics or merely increases local enzyme‐substrate concentration through physical tethering are warranted. In summary, this work provides the first mechanistic insight into FGF13‐mediated regulation of microtubule PTMs.

The study also has some potential limitations. First, RNA sequencing analysis revealed significant enrichment of genes related to mitochondrial damage and autophagy pathways in the SNS‐Cre; *Fgf13* ^fl/fl^ PTX‐treated group compared to the *Fgf13* ^fl/fl^ PTX‐treated group. Consistently, we also corroborated that FGF13 depletion ameliorates PIPNP by regulating mitophagy to reduce mitochondrial damage. Nevertheless, the pathogenesis of PIPNP is highly complex, encompassing a multitude of neural pathways and molecular mechanisms [7, 8]. Therefore, further investigation is warranted to identify additional signaling pathways that may be implicated in FGF13 regulation of PIPNP. Second, while our study establishes that FGF13 regulates mitophagy through the VASH1‐α‐tubulin axis in PIPNP, we did not investigate whether additional mitophagy‐related proteins (such as BNIP3, FUNDC1, or other autophagy receptors) may also contribute to mitochondrial quality control in this context. Future proteomic or interactome studies will be necessary to comprehensively map the protein network governing mitochondrial homeostasis during PIPNP. Third, FGF13 is also present in afferent fibers terminating in laminae I‐II of the spinal dorsal horn [44]. While our study focused on DRG somata, the FGF13‐VASH1‐microtubule axis likely operates in these nociceptive afferent terminals as well, given the presence of the necessary molecular components. Future studies using compartment‐specific manipulation will be needed to dissect the relative contributions of somatic vs. axonal mechanisms in PIPNP. Fourth, VASH1 forms an obligate heterodimer with SVBP to exert its tubulin carboxypeptidase activity [25, 28]. While our data demonstrate that FGF13 bridges VASH1 and α‐tubulin, we cannot exclude the possibility that FGF13 interacts with the VASH1‐SVBP heterodimer rather than VASH1 alone. Future studies using purified components will be required to determine whether FGF13 modulates the enzymatic activity of the VASH1‐SVBP complex or merely enhances its microtubule targeting. Fifth, although our Co‐IP and PLA data suggest physical interactions between FGF13 and VASH1, as well as between FGF13 and α‐tubulin, and molecular docking predicts potential binding sites, future studies are still needed to further validate whether these are direct protein‐protein interactions.

In summary, our study highlights for the first time that FGF13 regulates PIPNP by interacting with VASH1. FGF13 knockout reduces VASH1 binding to microtubules, thereby increasing microtubule tyrosination levels and subsequently activating mitophagy, which ultimately alleviates PTX‐induced mitochondrial damage and PIPNP. These findings provide novel insights suggesting that specifically targeting FGF13 and VASH1 in DRG neurons may represent a therapeutic strategy for PIPNP.

## 4 Experimental Section

### 4.1 Animals

All animal experiments were performed using protocols approved by the Laboratory Animal Ethical and Welfare Committee of Hebei Medical University (Shijiazhuang, China, Approval No. IACUC‐Hebmu‐P 2024146). Male C57BL/6J mice aged 8–16 weeks were maintained under standard specific pathogen‐free (SPF) conditions with controlled temperature (22‐24°C) and a 12/12‐h light/dark cycle, with ad libitum access to autoclaved food and sterile water. To generate DRG‐specific *Fgf13* conditional knockout mice, we crossed *Fgf13^flox/Y^* or *Fgf13^flox/flox^* mice with SNS‐Cre transgenic mice expressing Cre recombinase under the Nav1.8 promoter, enabling tissue‐specific deletion via Cre‐loxP recombination [45]. The Fgf13‐loxP mouse line was engineered through targeted insertion of loxP sites flanking exon 3 of the Fgf13 gene, a project conducted in collaboration with Beijing Biocytogen Co., Ltd. (Beijing, China). Mice with the genotype *Fgf13^fl/Y^*; SNS‐Cre (designated as *Fgf13^−/Y^*) were classified as conditional knockout mice. Genomic DNA extracted from tail biopsies was used for genotyping following established protocols [46]. The primer sequences and cycling conditions were listed in Table S1.

### 4.2 Animal Model of PIPNP and Treatment

The model of PIPNP was established as previously described [47]. In brief, The administered drug was prepared by dissolving PTX (MedChemExpress, Newark, NJ, USA; HY‐B0015) in Dimethyl sulfoxide (DMSO; Solarbio, Beijing, China; D8371) and then diluting it with sterile corn oil (MedChemExpress, Newark, NJ, USA; HY‐Y1888) to achieve a final concentration of 10% DMSO. Mice received PTX at a dose of 8 mg/kg bodyweight via intraperitoneal (i.p.) injection on days 0, 2, 4, and 6, resulting in a cumulative dose of 32 mg/kg over the treatment period. The vehicle control group received an injection (i.p.) of the solvent mixture (10% DMSO in 90% corn oil) at a volume of 4 mL/kg, following the identical injection schedule.

Autophagy inhibition in vivo was achieved by intraperitoneal injection of chloroquine (CQ) (60 mg/kg/day; Sigma‐Aldrich, St. Louis, MO, USA;C6628) or Baf A1 (0.3 mg/kg/day; Sigma‐Aldrich, St. Louis, MO, USA;B1793) on the 11th, 12th, and 13th days.

### 4.3 Behavioral Analysis

Mechanical pain sensitivity was assessed through Von Frey filament testing [47]. Prior to evaluation, mice were acclimated for 30 min in individual testing chambers with mesh flooring. Graduated filaments ranging from 0.008 to 1.4 g were applied perpendicularly to the hind paw plantar surface to determine withdrawal thresholds. Each filament application was maintained for approximately 3 s, with three consecutive trials conducted at 10 s intervals per filament strength. The testing protocol involved progressively increasing filament stiffness until a positive withdrawal response was observed. For the thermal stimulation, experimenters blinded to the experimental conditions assessed the mice using thermal stimulation. Thermal nociception was assessed at baseline (day 0, prior to initial PTX administration) and subsequently on post‐treatment days 1, 3, 5, 7, and 14. Briefly, mice were acclimated in individual testing chambers placed on a glass platform for 30 min prior to behavioral assessment. We used the PL‐200 Thermal Nociception Analyzer (Chengdu Taimeng Software, Chengdu, Sichuan, China) with the light intensity adjusted to 10% of the maximum output. The radiant heat stimulus was precisely targeted to the central plantar region of each hind paw, and paw withdrawal latency was measured. Three trials were conducted on the right hind paw at 5‐min intervals, and the mean withdrawal latency was calculated by averaging all three measurements.

### 4.4 Primary Cell Cultures and Treatments

DRG neurons were isolated and cultured following established protocols [48]. Briefly, L3–L5 DRGs were dissected from adult mice (8‐16 weeks old) and placed in Hank's Balanced Salt Solution supplemented with collagenase Type II (2.5 mg/mL, Worthington Biochemical, USA; LS004176) and Dispase (7.5 mg/mL, Sigma‐Aldrich, St. Louis, MO, USA; 04942078001), followed by incubation at 37°C for 30 min. The DRGs were gently washed and resuspended in DMEM‐based (Thermo Fisher Scientific, Waltham, MA, USA; 11995065) culture medium supplemented with 10% fetal bovine serum (FBS) (Thermo Fisher Scientific, Waltham, MA, USA; 10270‐106), 1% penicillin, and streptomycin (Sevenbio, Beijing, China; 15140122).

### 4.5 Plasmid and Small RNA Interference

The expression plasmids for Flag‐tagged VASH1, Flag‐tagged TTL, and Myc‐FGF13 (Myc‐FGF13‐FL), along with its variants including Myc‐FGF13 (∆1‐37), Myc‐FGF13 (∆1‐37), Myc‐FGF13 (∆38‐75), Myc‐FGF13 (∆76‐99), Myc‐FGF13 (∆100‐145) and Myc‐FGF13 (∆146‐192) were purchased from GeneChem Co., Ltd. (Shanghai, China). The plasmids were transfected into HEK‐293T cells using E‐Trans DNA reagent (GeneChem, Shanghai, China; REVG007) according to the standard protocol. The siRNA of KIF1A or negative control siRNA was obtained from GeneChem Co., Ltd. (Shanghai, China). The siRNA sequence was as follows: KIF1A forward primer: GUGUGGAGCUAAAGAAGAATT, KIF1A reverse primer: UUCUUCUUUAGCUCCACACTT. siRNA transfection was conducted using RNAFit reagent (Hanbio Technology, Shanghai, China; HB‐RF‐1000).

### 4.6 RNA Sequencing and Bioinformatics Analysis

The library construction and sequencing were performed by Applied Protein Technology Co., Ltd. (Shanghai, China). Total RNA was extracted from the L3‐L5 DRG tissue using Trizol Reagent (Thermo Fisher Scientific, Waltham, MA, USA; 15596026CN) according to the manufacturer's instructions. RNA samples were detected based on the A260/A280 absorbance ratio with a Nanodrop ND‐2000 system (Thermo Scientific, USA), and the RIN of RNA was determined by an Agilent Bioanalyzer 4150 system (Agilent Technologies, CA, USA). Paired‐end libraries were prepared using a ABclonal mRNA‐seq Lib Prep Kit (ABclonal, China) following the manufacturer's instructions. Sequencing was performed with an Illumina Novaseq 6000 instrument. The data generated from Illumina platform was used for bioinformatics analysis. FeatureCounts was used to count the reads numbers mapped to each gene. And then fragments per kilobase of exon per million reads mapped (FPKM) of each gene was calculated based on the length of the gene and reads count mapped to this gene. Differentially expressed RNAs between *Fgf13* ^fl/fl^ +PTX and SNS‐Cre; *Fgf13* ^fl/fl^ +PTX mice with |log2FC|>1 and *p* value <0.05 were considered statistically significant. KEGG enrichment pathway analysis and Gene Ontology (GO) functional enrichment analysis and were performed using the clusterProfiler R software package.

### 4.7 Western Blot Analysis

Protein levels of FGF13, VASH1, p62, LC3, Beclin 1, Pink 1, Parkin, TOMM20, KIF1A, FLAG, MYC, Tyr‐tubulin, Detyr‐tubulin, α‐tubulin and GAPDH were examined via Western blotting. In brief, mice L3‐L5 DRG tissue samples were lysed with RIPA buffer (Sevenbio, Beijing, China; SW104‐02) supplemented with protease inhibitor cocktail (MedChemExpress, Newark, NJ, USA; HY‐K0010) to obtain total protein. Lysates were centrifuged at 12 000 rpm/min for 30 min at 4°C, and the supernatant was collected. The concentrations of protein in the supernatant were measured using a bicinchoninic acid (BCA) kit (Solarbio, Beijing, China; PC0020). Equal amounts of protein samples and Protein Ladder (Thermo Fisher Scientific, Waltham, MA, USA; 26617; Biotides, Beijing, China; WB1902) were subjected to 10% SDS‐PAGE (Sevenbio, Beijing, China; SW143‐02), 12% SDS‐PAGE (Sevenbio, Beijing, China; SW145‐02), or FuturePAGE 4–20% 12 Wells (ACE Biotechnology, Shanghai, China; ET12420Gel), and transferred to PVDF membranes. The membranes were blocked with 5% non‐fat milk in TBST at room temperature for 2 h and then respectively incubated with primary antibodies against FGF13 (1:500, Thermo Fisher Scientific, Waltham, MA, USA; 3D299A37), VASH1 (1:500, Santa Cruz Biotechnology, Dallas, TX, USA; sc‐365541; 1:500, Immunoway, Chengdu, China; YT4853), p62 (1:10,000, Abcam, Cambridge, MA, USA; ab109012), LC3 (1:1000, Cell Signaling Technology, Boston, MA, USA; 12741S), FLAG (1:1000, zen‐bioscience, Chengdu, China; R24091), MYC (1:2000, Proteintech, Wuhan, China; 60003‐2‐Ig), Tyr‐tubulin (1:1000, Sigma–Aldrich, St. Louis, MO, USA; MAB1864‐I), Detyr‐tubulin (1:1000, Abcam, Cambridge, MA, USA; ab254154), α‐tubulin (1:10 000, Proteintech, Wuhan, China; 11224‐1‐AP; 1:20 000, Proteintech, Wuhan, China; 66031‐1‐Ig), Beclin 1 (1:1000, Proteintech, Wuhan, China; 11306‐1‐AP), Pink 1 (1:500, Abmart, Shanghai, China; PK05715S), Parkin (1:1000, Proteintech, Wuhan, China; 14060‐1‐AP), TOMM20 (1:1000, Abcam, Cambridge, MA, USA; ab186735), KIF1A (1:1000, Abcam, Cambridge, MA, USA; ab271047), and GAPDH (1:5000, zen‐bioscience, Chengdu, China; 200306–7E4; 1:10 000, zen‐bioscience, Chengdu, China; R380626) at 4°C overnight. The samples were then incubated for 2 h at room temperature with fluorophore‐conjugated Goat anti‐Mouse (1:10 000, LI‐COR Biosciences, Lincoln, NE, USA; 962–68070) and Goat anti‐Rabbit (1:10,000, LI‐COR Biosciences, Lincoln, NE, USA; 926–32211) secondary antibodies. The emitted light was detected and analyzed using an Odyssey gel imaging system (LI‐COR, Lincoln, NE, USA) and relative target band intensity was normalized to GAPDH on ImageJ (version 1.8.0; U.S. National Institutes of Health, Bethesda, MD, USA, https://imagej.net/ij/index.html).

### 4.8 Immunofluorescence Assay

L3–L5 DRG tissues were immersion‐fixed in 4% PFA (Sevenbio, Beijing, China; SI101‐01) at 4°C for 12 h, followed by cryoprotection through graded sucrose solutions (10% and 30%) with overnight incubation at 4°C, and finally equilibrated in a 1:1 mixture of 30% sucrose and OCT (Sakura Finetek, Tokyo, Japan; 4583) compound overnight at 4°C. Following cryoprotection, tissues were embedded in OCT frozen at −80°C, and sectioned at 10 µm thickness using a cryostat microtome (Leica Biosystems, Wetzlar, Germany). Tissue sections were washed with PBS for 10 min, permeabilized at 37°C for 60 min using a solution containing 3% BSA (BioFroxx GmbH, Eching, Germany; 4240GR100) and 0.3% Triton X‐100 (Sigma‐Aldrich, St. Louis, MO, USA; 9036‐19‐5), and subsequently blocked with blocking‐grade normal goat serum (Zhongshan Goldenbridge Biotechnology, Beijing, China; ZLI‐9056) at 37°C for 30 min. The sections were incubated with different primary antibodies: rabbit anti‐FGF13 (1:200, Yenzym Biotechnology, Wuhan, Hubei, China; YZ5318), mouse anti‐α‐tubulin (1:1000, Proteintech, Wuhan, China; 66031‐1‐Ig), rabbit anti‐KIF1A (1:50, Abcam, Cambridge, MA, USA; ab271047), mouse anti‐Tyr‐tubulin (1:250, Sigma–Aldrich, St. Louis, MO, USA; MAB1864‐I), mouse anti‐VASH1 (1:400, Santa Cruz Biotechnology, Dallas, TX, USA; sc‐365541), rabbit anti‐GFAP (1:100, Cell Signaling Technology, Boston, MA, USA; 12389) and rabbit anti‐Nav1.8/SCN10A (1:100, Abcam, Cambridge, MA, USA; ab93616) overnight at 4°C. The secondary antibodies were Goat Anti‐Mouse IgG H&L (Alexa Fluor 488) (1:200, Abcam, Cambridge, MA, USA; ab150113) and Goat Anti‐Rabbit IgG H&L (Alexa Fluor 647) (1:200, Abcam, Cambridge, MA, USA; ab150079) diluted in the Antibody dilution buffer (Zhongshan Goldenbridge Biotechnology, Beijing, China; ZLI‐9030) at RT for 90 min. Finally, the specimens were mounted with anti‐fade mounting medium containing DAPI (Sevenbio, Beijing, China; SI103‐12) to preserve fluorescence signals and counterstain nuclei. The samples were observed under a laser scanning confocal microscope (Leica, Wetzlar, Germany; TCS SP8). To ensure quantitative comparability of immunofluorescence signals, all images were acquired under standardized microscopy parameters (including exposure time, gain, and light intensity) maintained consistently throughout the imaging sessions.

### 4.9 Proximity Ligation Assay (PLA)

DRG neurons were seeded in 24‐well plates on coverslips.After cultured 24 h, cells were fixed, permeabilized, blocked, and incubated with primary rabbit anti‐FGF13 (1:200, Yenzym Biotechnology, Wuhan, Hubei, China; YZ5318), mouse anti‐α‐tubulin (1:1000, Proteintech, Wuhan, China; 66031‐1‐Ig), rabbit anti‐α‐tubulin (1:500, Proteintech, Wuhan, China; 11224‐1‐AP and mouse anti‐VASH1 (1:400, Santa Cruz Biotechnology, Dallas, TX, USA; sc‐365541) at 4°C overnight. The following experimental steps were performed according to the manufacturer's instructions provided by Sigma‐Aldrich for the Duolink In Situ Red Starter Kit Mouse/Rabbit (Sigma–Aldrich, St. Louis, MO, USA; DUO92101). For WT, SNS‐Cre; *Fgf13* ^fl/fl^ and AAV‐shVASH1 mice, DRG tissues were incubated with primary antibodies at 4°C overnight. For negative controls, we performed single‐antibody controls by incubating samples with only one primary antibody (either anti‐FGF13 or anti‐VASH1) together with secondary antibodies and PLA probes. Specifically, control samples were processed identically to experimental samples except that one of the two primary antibodies was replaced with antibody dilution buffer. These single‐antibody control samples showed no detectable PLA signals, confirming that both primary antibodies are required for PLA signal generation and validating the specificity of the observed FGF13‐VASH1, VASH1‐α‐tubulin, and FGF13‐α‐tubulin protein‐protein interactions. Finally, coverslips were mounted onto slides and imaged using a laser scanning confocal microscope (Leica, Wetzlar, Germany; TCS SP8). Finally, coverslips were mounted onto slides and imaged using a laser scanning confocal microscope (Leica, Wetzlar, Germany; TCS SP8).

### 4.10 Quantitative Real‐Time PCR Analysis

Total RNA was extracted from mouse L3‐L5 DRG tissues using Trizol Reagent (Thermo Fisher Scientific, Waltham, MA, USA; 15596026CN). First‐strand cDNA synthesis was performed with total RNA as template using the UnionScript First‐strand cDNA Synthesis Mix (Genesand Biotech Co, Beijing, China; SR511). Quantitative reverse transcription PCR was carried out in a 25 µL reaction volume containing 1 µL forward and reverse primers, 2 µL of cDNA template, 6 µL DEPC‐treated water and 10 µL 2×GSAntiQ qPCR SYBR Fast Mix(Universal) (Genesand Biotech Co, Beijing, China; SQ410) on the QuantStudioTM 5 Real‐Time PCR Instrument (Thermo Fisher Scientific, Waltham, MA, USA; A28134). The expression level of targeted genes was normalized to that of GAPDH, which was regarded as an endogenous internal control. Primer sequences used in this study are listed in Table S2.

### 4.11 Assessment of Lysosome and Mitochondria Colocalization

Mitophagy in DRG neurons was monitored using a mitophagy detection kit (Dojindo Molecular Technologies, Kumamoto, Japan; MD01) containing Mtphagy Dye and Lyso Dye. Briefly, L3‐L5 DRG neurons were seeded in 24‐well cell culture plates and cultured overnight. Cells were washed twice with Hanks’ HEPES buffer and then incubated with 100 nM Mtphagy Dye for 30 min at 37°C. After washing again, cells were incubated with 50 nM Lyso Dye for 30 min at 37°C. Following final washes, live‐cell imaging was immediately performed using a confocal fluorescence microscope (Leica, Wetzlar, Germany; TCS SP8). Mitochondrial‐lysosomal co‐localization was quantified using ImageJ (version 1.8.0; U.S. National Institutes of Health, Bethesda, MD, USA, https://imagej.net/ij/index.html).

### 4.12 Measurement of the ΔΨM

ΔΨM was assessed using a JC‐1 assay kit (Beyotime, Shanghai, China; C2006). JC‐1 is a mitochondrial potential indicator that exists either as a green fluorescent monomer at depolarized membrane potentials or as a red fluorescent J‐aggregate at hyperpolarized membrane potentials. L3‐L5 DRG neurons were seeded in 24‐well plates and cultured overnight. After removing culture medium, cells were incubated with 1 mL JC‐1 working solution (prepared per kit instructions) at 37°C for 20 min in the dark. Following two washes with 1 × JC‐1 staining buffer, cells were maintained in the buffer for imaging. Live‐cell imaging was immediately performed using a confocal fluorescence microscope (Leica, Wetzlar, Germany; TCS SP8) with 490 nm excitation/530 nm emission (green monomer) and 525 nm excitation/590 nm emission (red J‐aggregates). The J‐aggregate/monomer fluorescence intensity ratio was quantified in ImageJ (version 1.8.0; U.S. National Institutes of Health, Bethesda, MD, USA, https://imagej.net/ij/index.html) to assess ΔΨm changes.

### 4.13 Measurement of Mitochondrial ROS Production

Intracellular ROS levels were quantified using a DCFH‐DA assay kit (Solarbio, Beijing, China; CA1410). L3‐L5 DRG neurons cultured overnight in 24‐well plates were washed twice with pre‐warmed phosphate‐buffered saline (PBS, pH 7.4). Cells were loaded with 10 µM DCFH‐DA (diluted in serum‐free medium) for 30 min at 37°C in the dark. After three gentle washes with serum‐free culture medium, live‐cell was observed with a confocal fluorescence microscope (Leica, Wetzlar, Germany; TCS SP8) in FITC channel (ex/em 488/525 nm) and the mean fluorescence intensity was quantified after background subtraction using ImageJ (version 1.8.0; U.S. National Institutes of Health, Bethesda, MD, USA, https://imagej.net/ij/index.html).

### 4.14 Transmission Electron Microscopy (TEM)

The L3‐L5 DRG tissue was fixed with 2.5% glutaraldehyde for 48 h. After overnight fixation, samples were postfixed in 1% osmium tetroxide, dehydrated through graded concentrations of ethanol, infiltrated, and embedded in Epon 812 at 60°C for 48 h. Ultrathin sections were cut with a diamond knife, mounted on formvar‐coated slot grids, and then stained with 2% uranyl acetate and lead citrate for 15 min, respectively. The images were taken with a HT7700 model Hitachi TEM (Hitachi, Tokyo, Japan).

### 4.15 Co‐Immunoprecipitation (Co‐IP)

Either DRG tissues or HEK‐293T cells were lysed in 1 mL IP lysis buffer (Beyotime, Shanghai, China; P0013) supplemented with phosphatase inhibitor cocktail (MedChemExpress, Newark, NJ, USA; HY‐K0021), and protease inhibitor cocktail (MedChemExpress, Newark, NJ, USA; HY‐K0010). The IP lysis buffer composition included 20 mM Tris (pH 7.5), 150 mM NaCl, 1% Triton X‐100, sodium pyrophosphate (2 mM), β‐glycerophosphate (10 mM), EDTA (1 mM), and leupeptin (10 µg/mL). After lysis at 4°C for 20 min, supernatants were collected by centrifugation at 12 000 rpm for 15 min at 4°C, and protein concentration was measured using a BCA assay (Solarbio, Beijing, China; PC0020). For immunoprecipitation, 6–10 µg of either primary antibody or control IgG was incubated with 16–20 µL Protein A+G magnetic beads (Beyotime, Shanghai, China; P2108) for 2 h at room temperature with rotation. After washing the beads to remove unbound antibody, 1,000 µg of protein lysate was added to the beads and incubated overnight at 4°C with rotation. The beads were then collected magnetically, washed three times with ice‐cold IP lysis buffer, and eluted in 1× protein loading buffer at 95°C for 5 min. Eluted proteins were analyzed by Western blotting as previously described.

### 4.16 Adeno‐Associated Virus (AAV) Delivery

An AAV system was used to deliver FGF13‐wt, FGF13‐mut, VASH1, or their respective control vectors to treated mice. Recombinant AAV‐PHP.S vectors for neuronal expression‐including AAV‐hSyn‐FGF13 (for wild‐type FGF13 overexpression), AAV‐hSyn‐FGF13‐mut (for mutant FGF13 overexpression), AAV‐hSyn‐VASH1 (for VASH1 overexpression in DRG neurons), and AAV‐hSyn‐shKIF1A (for KIF1A knockdown in DRG neurons)‐were purchased from GeneChem Co., Ltd. (Shanghai, China). Each mouse received 5 × 10^1^ ^1^ viral genomes (vg) of AAV via intrathecal injection administered by lumbar puncture over 4 weeks. Subsequently, AAV transfection efficiency was verified by Western blotting.

### 4.17 Protein‐Protein Docking Studies

The FGF13‐VASH1 binding mode was analyzed by molecular docking. We downloaded the protein sequences of FGF13 (PDB ID: 3HBW), VASH1‐SVBP complex (PDB ID: 6J4U) and α‐tubulin (PDB ID: 3J8X) from the UniProt database (https://www.uniprot.org). The 3D structure models of FGF13 and VASH1‐SVBP, and FGF13, VASH1 and α‐tubulin were built by AlphaFold2 respectively. We performed molecular docking using the HDOCK program and selected the structure with the best docking score as the standard result for subsequent interaction analysis. The docking scores were calculated based on the ITScorePP or ITScorePR iterative scoring function. PyMOL software was used to visualize the docking results of the members with the highest scores.

### 4.18 Statistical Analysis

All experiments were repeated for at least 3 biological replicates. Statistical analyses were performed by using GraphPad Prism 9.5.0 (GraphPad Software Inc., La Jolla, CA, USA), with data expressed as mean ± SEM (standard error of the mean). For comparisons between two groups of equal sample size, an unpaired two‐tailed Student's t‐test was performed. For comparisons of more than two groups, a one‐way ANOVA followed by Tukey's multiple comparisons test was used. Two‐way ANOVA followed by Tukey's post hoc test was used to test the difference among groups with various factors.For multiple comparison testing, a one‐way analysis of variance (ANOVA) accompanied by Tukey's post hoc test was used. Differences were considered significant when *p* was <0.05, <0.01 or <0.001.

## Author Contributions

D.Y.M., W.Y.D., and L.S.M. contributed equally to this work. D.Y.M. designed the research and drafted the manuscript. D.Y.M., W.Y.D., and L.S.M. performed experiments, analyzed the data and created all the figures. D.Y.M., W.Y.D., L.S.M., D.Z.S., Z.K.X., G.X.H., L.X.Y., Y.R.X., Z.Y.Y., C.S.Y., and W.C. discussed the results and revised the manuscript. W.C. supervised the research design and revised the final version of the manuscript. All authors reviewed and approved the manuscript.

## Conflicts of Interest

The authors declare no conflicts of interest.

## Supporting information




**Supporting File**: advs76106‐sup‐0001‐SuppMat.docx.

## Data Availability

The data that support the findings of this study are available on request from the corresponding author. The data are not publicly available due to privacy or ethical restrictions.
