# Supplementary material for: FGF13 Deficiency Ameliorates Paclitaxel‐Induced Neuropathic Pain by Inhibiting VASH1‐Mediated Microtubule Detyrosination to Promote Mitophagy
Source: Adv Sci (Weinh). 2026 Jun 18:e20995. Online ahead of print. doi: 10.1002/advs.202520995 (PMC13336967; doi:10.1002/advs.202520995)
Supplement: Supplementary file 1 — Supporting File: advs76106‐sup‐0001‐SuppMat.docx. [file ADVS-9999-e20995-s001.docx]

Supporting Information

FGF13 Deficiency Ameliorates Paclitaxel-Induced Neuropathic Pain by Inhibiting VASH1-Mediated Microtubule Detyrosination to Promote Mitophagy

***Authors***

Yiming Dong, Yidan Wang, Simeng Lv, Zishan Dong, Kaixi Zhi, Xiuhua Guo, Xuyan Li, Ruoxi Yu, Yiyi Zhang, Siyuan Cheng, and Chuan Wang^*^

**AFFILIATIONS**

Y. Dong, Y. Wang, S. Lv, K. Zhi, X. Guo, C. Wang

Key Laboratory of New Drug Pharmacology and Toxicology, Key Laboratory of Neural and Vascular Biology, Ministry of Education, Hebei Medical University, Shijiazhuang 050017, China
E-mail: [wangchuan@hbu.edu.cn](mailto:wangchuan@hbu.edu.cn)

Z. Dong

Hebei Key Laboratory of Critical Disease Mechanism and Intervention, Department of Pathophysiology, Neuroscience Research Center, Hebei Medical University, Shijiazhuang 050017, China

X. Li, R. Yu

College of Basic Medicine, Hebei Medical University, Shijiazhuang 050017, China

Y. Zhang, C. Wang

Affiliated Hospital of Hebei University, Clinical Medical College, Hebei University, Baoding 071000, China

S. Cheng

College of Basic Medical Sciences, Hebei University, Baoding 071002, China

C. Wang

Department of Pharmacology , Hebei Medical University, Shijiazhuang 050017, China

C. Wang

College of Pharmaceutical Sciences, Key Laboratory of Medicinal Chemistry and Molecular Diagnosis, Ministry of Education, College of Life Sciences, Hebei University, Baoding 071002, China

**This file includes the following subsections:**

Figure S1-S17;

Table S1-S2.

**Supplementary Figures**


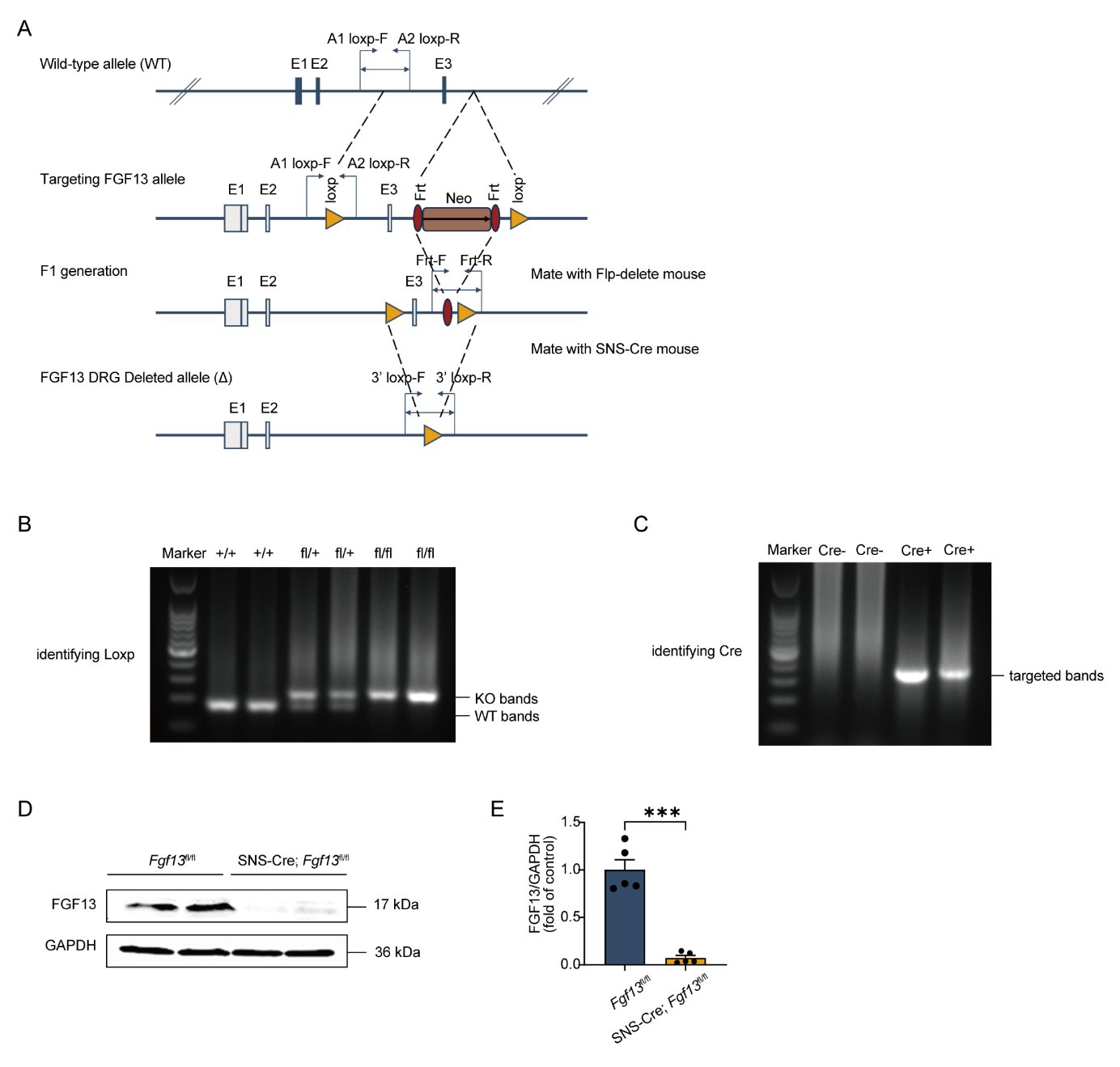


**Figure S1. Generation of *Fgf13* conditional knockout mice using Cre-loxP system.**

**(A)** A schematic diagram of *Fgf13* conditional knockout mice generation using Cre-loxP system. **(B-C)** Representative PCR gel images of genotyped mice. The target band for Cre was ~350 bp; the target band for FGF13 knockout (KO) mice (241 bp) was larger in size than that for wild-type (WT) mice (183 bp), as shown in the panel. **(D-E)** Western blotting and quantitative analysis of FGF13 expression level in DRG tissues of *Fgf13*^fl/fl^ and SNS-Cre; *Fgf13*^fl/fl^ mice (n = 5 per group). Data presented as mean ± SEM. ****p* < 0.001. Two-tailed unpaired Student’s t-test was used in E. FGF13, fibroblast growth factor 13; KO, knockout; WT, wild type.


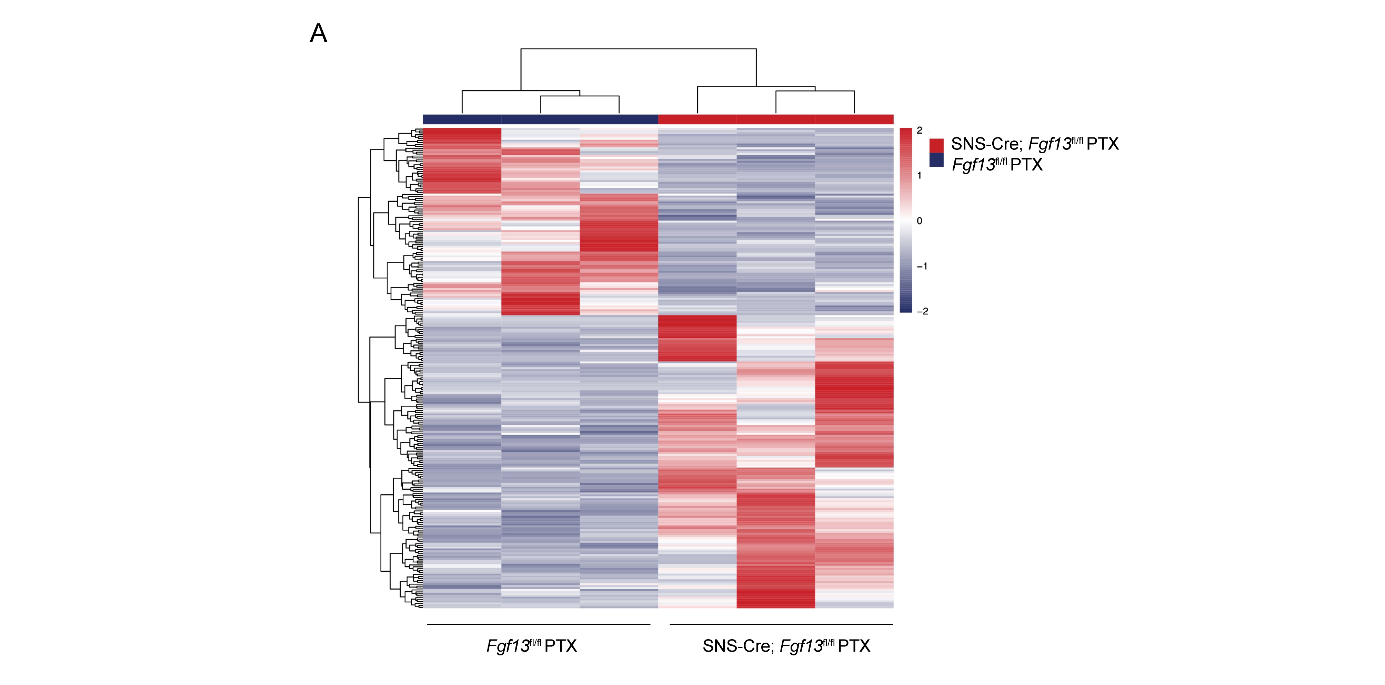


**Figure S2. Transcriptional profiling of DRG tissues from *Fgf13*^fl/fl^ and SNS-Cre; *Fgf13*^fl/fl^ mice.**

**(A)** Hierarchical clustering heatmap of dysregulated transcripts in DRG tissues of *Fgf13*^fl/fl^ and SNS-Cre; *Fgf13*^fl/fl^ mice ((n = 3 per group). PTX, paclitaxel.


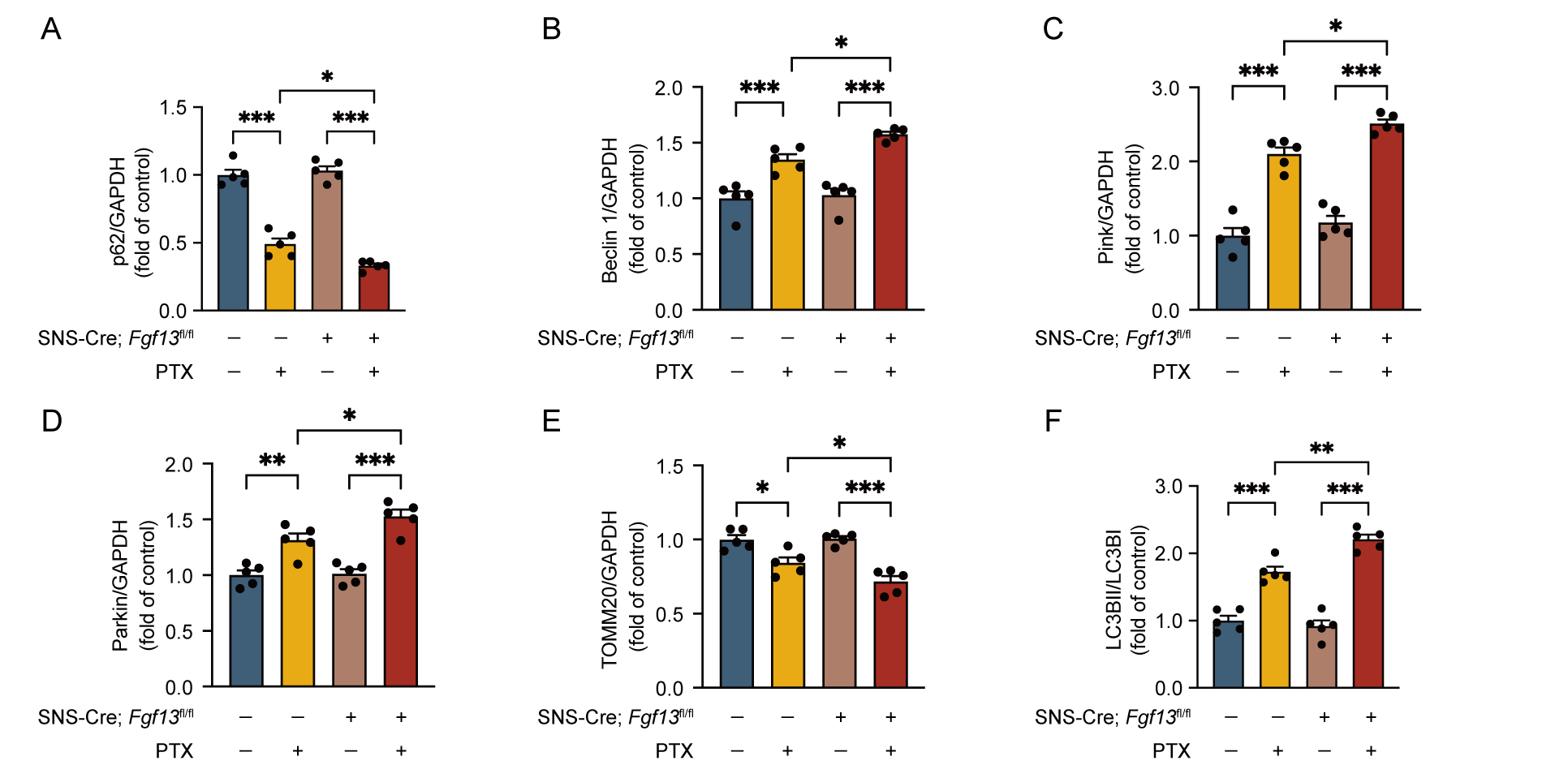


**Figure S3. FGF13 depletion enhances mitophagy activation in DRG neurons during PIPNP.**

**(A-F)** Quantitative analysis of mitophagy and autophagy markers in DRG tissues from *Fgf13*^fl/fl^ and SNS-Cre; *Fgf13*^fl/fl^ mice with or without PTX treatment. (A) p62, (B) Beclin1, (C) PINK1, (D) Parkin, (E) TOMM20, and (F) LC3-II/LC3-I ratio. Data are normalized to GAPDH and presented as fold of control. Data represent mean ± SEM; **p* < 0.05, ***p* < 0.01, ****p* < 0.001. Two-way ANOVA followed by Tukey’s multiple comparisons test was used in A-F. PTX, paclitaxel.


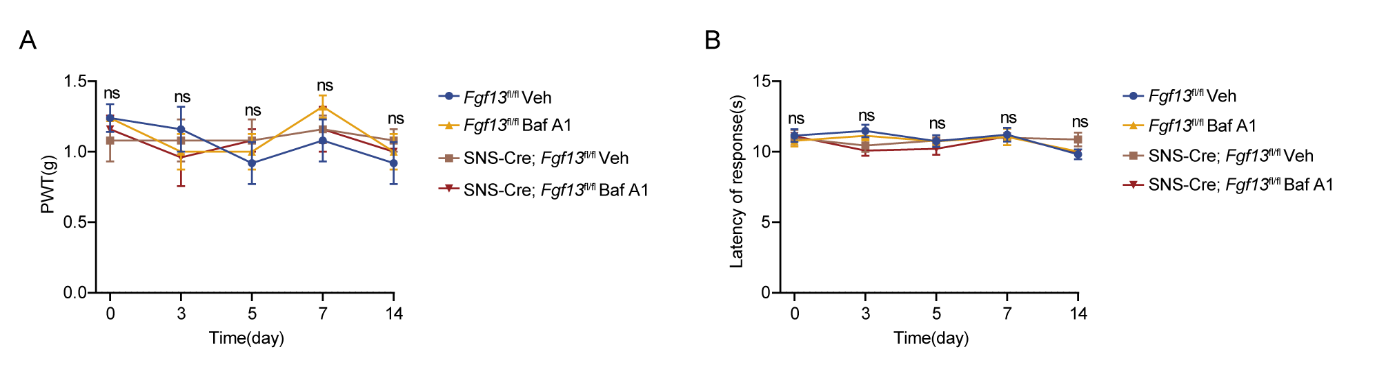


**Figure S4. FGF13 depletion ameliorates PIPNP through enhanced mitophagy. Related to Figure 3H, I.**

**(A)** Mechanical withdrawal thresholds and **(B)** thermal withdrawal latencies in *Fgf13*^fl/fl^ and SNS-Cre; *Fgf13*^fl/fl^ mice under physiological conditions, with or without intrathecal Baf A1 treatment (n = 8 per group). Data presented as mean ± SEM. ns, not significant. Two-way ANOVA followed by Tukey’s multiple comparisons test was used in A, B. PWT, paw withdrawal mechanical threshold; PWL, paw withdrawal thermal latency. Baf A1, bafilomycin A1.


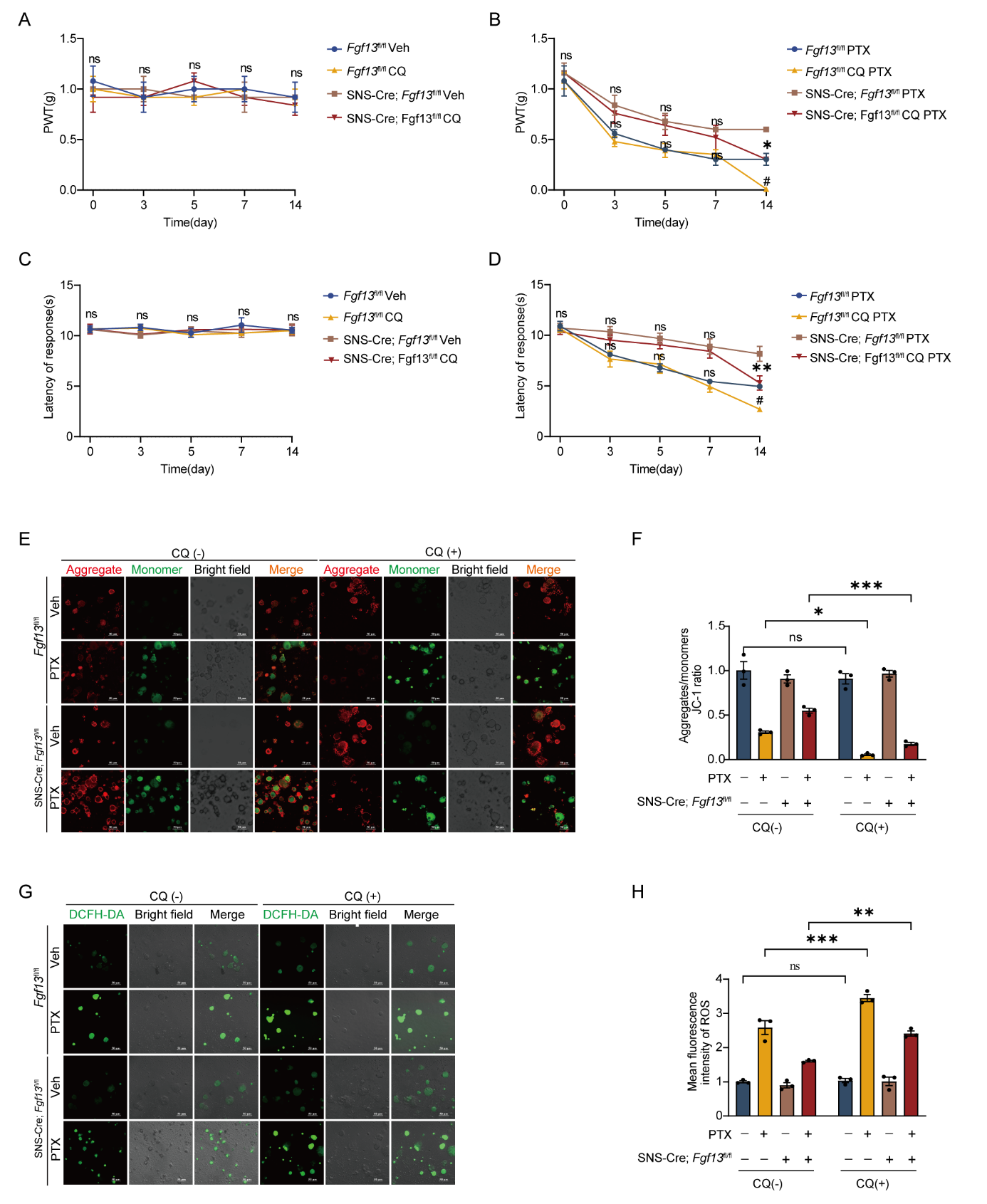


**Figure S5. FGF13 depletion ameliorates PIPNP by reducing mitochondrial damage through enhanced mitophagy.**

**(A)** Mechanical withdrawal thresholds and **(C)** thermal withdrawal latencies in *Fgf13*^fl/fl^ and SNS-Cre; *Fgf13*^fl/fl^ mice under physiological conditions, with or without intrathecal Baf A1 treatment (n = 8 per group). **(B)** Mechanical withdrawal thresholds and **(D)** thermal withdrawal latencies in *Fgf13*^fl/fl^ and SNS-Cre; *Fgf13*^fl/fl^ mice under PIPNP conditions, with or without intrathecal CQ treatment (n = 8 per group). **(E)** The mitochondrial membrane potentials (ΔΨM) of DRG neurons were determined by JC-1 staining. Scale bar = 50 μm. **(F)** The ratio of JC-1 aggregate to JC-1 monomer was compared in the bar graph (n = 3 per group). **(G)** Representative confocal images of DCFH-DA (green) staining in DRG neurons from *Fgf13*^fl/fl^ and SNS-Cre; *Fgf13*^fl/fl^ mice. **(H)** Quantification of relative ROS fluorescence intensity in DRG neurons (n = 3 per group). Data presented as mean ± SEM. **p* < 0.05, ***p* < 0.01, ****p* < 0.001. ^#^*p* < 0.05. ns, not significant. Two-way ANOVA followed by Tukey’s multiple comparisons test was used in A, B, C, D, F and H. CQ, chloroquine.


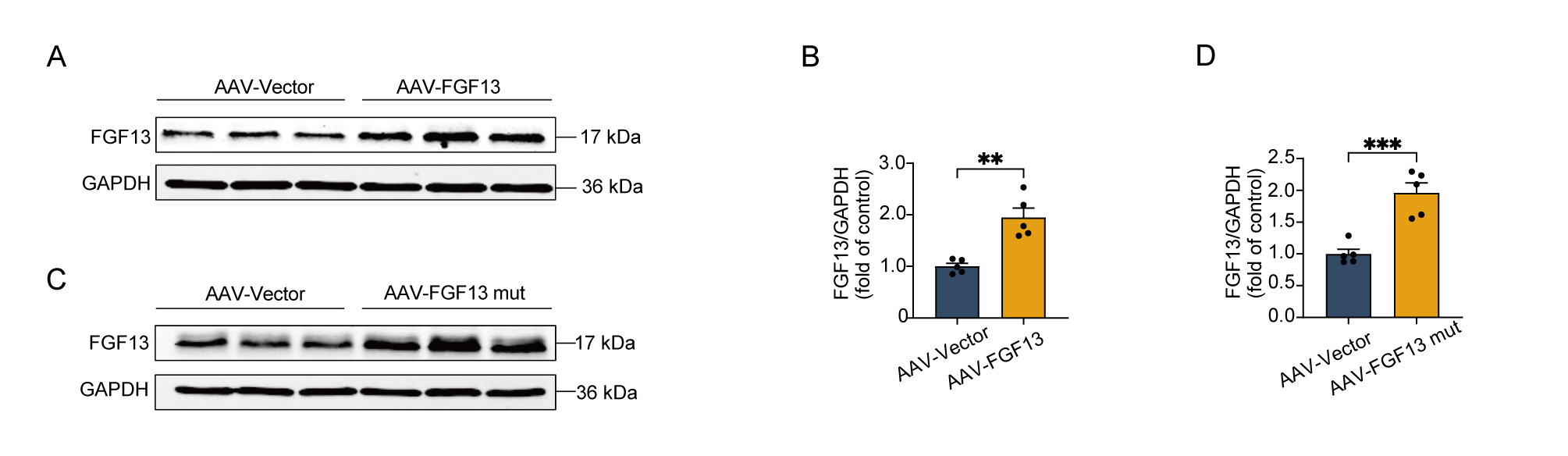


**Figure S6. Verification of the effects of AAV-mediated overexpression of FGF13 and FGF13 mut in mouse DRG tissues.**

**(A-B)** Western blotting and quantitative analysis of FGF13 expression in DRG tissues from mice injected with AAV-hSyn-Vector or AAV-hSyn-FGF13-WT (n = 5 per group). **(C-D)** Western blotting and quantitative analysis of FGF13 expression in DRG tissues from mice injected with AAV-hSyn-Vector or AAV-hSyn-FGF13-Mut (n = 5 per group). The protein level was standardized by GAPDH. Data represent mean ± SEM. ***p* < 0.01, ****p* < 0.001. Two-tailed unpaired Student’s t-test was used in B, D.


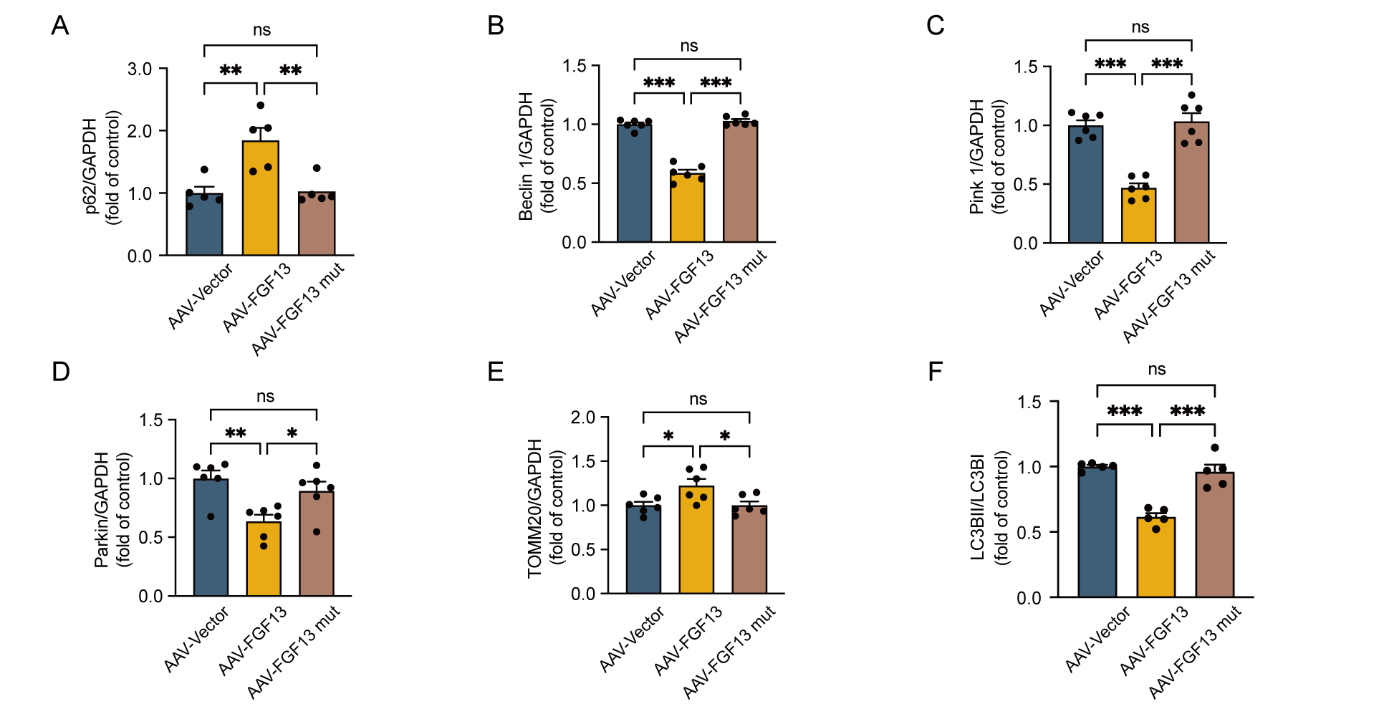


**Figure S7. FGF13 suppresses mitophagy via microtubule interaction.**

**(A-F)** Quantitative analysis of mitophagy and autophagy markers in DRG tissues from control mice and mice overexpressing FGF13 or FGF13 mutant under PIPNP conditions. (A) p62, (B) Beclin1, (C) PINK1, (D) Parkin, (E) TOMM20, and (F) LC3-II/LC3-I ratio. Data are normalized to GAPDH and presented as fold of control. Data represent mean ± SEM. **p* < 0.05, ***p* < 0.01, ****p* < 0.001. ns, not significant. One-way ANOVA followed by Tukey’s multiple comparisons test was used in A-F.


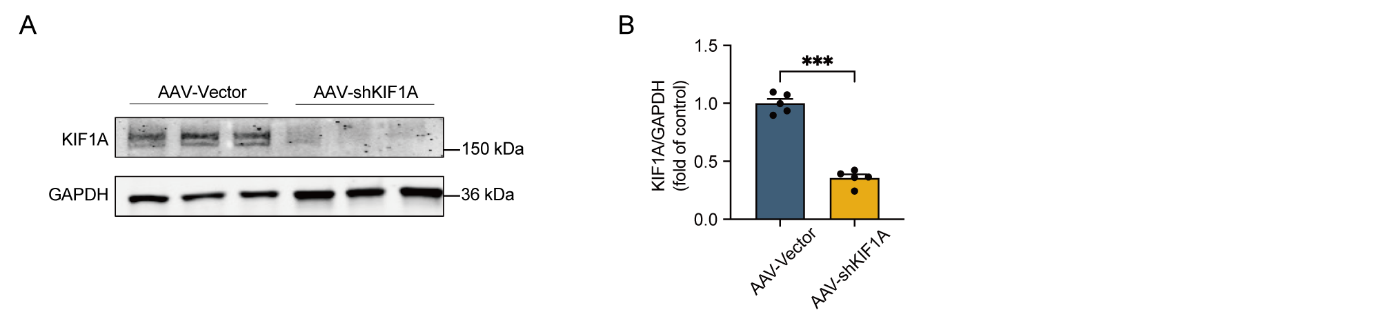


**Figure S8. Knockdown efficiency of KIF1A.**

**(A-B)** Western blotting and quantitative analysis of KIF1A expression in DRG tissues from mice injected with AAV-Vector or AAV-KIF1A (n = 5 per group). Data represent mean ± SEM. ****p* < 0.001. Two-tailed unpaired Student’s t-test was used in B. KIF1A, kinesin family member 1A.


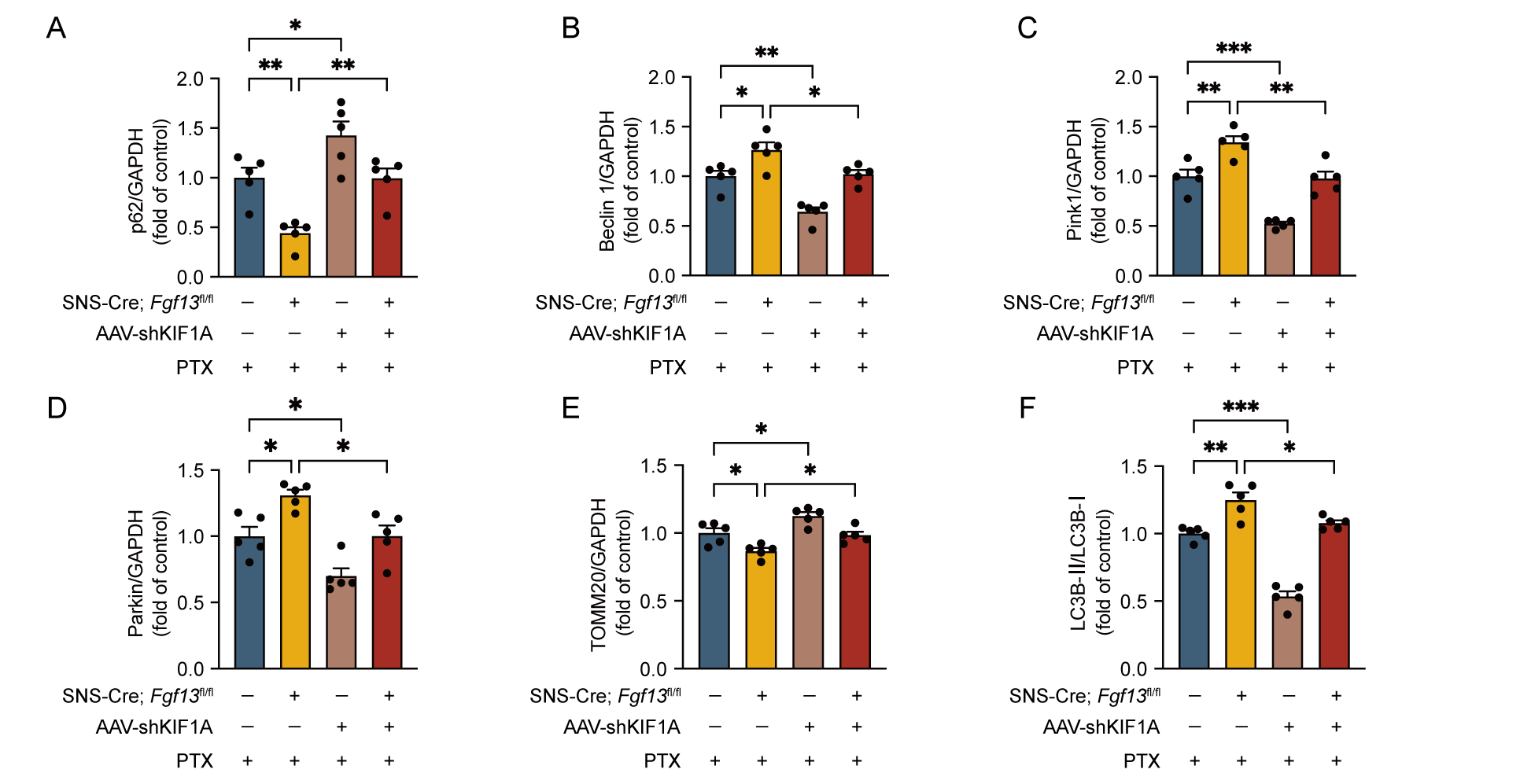


**Figure S9. FGF13 deficiency promotes mitophagy through KIF1A-dependent mechanism in PIPNP.**

**(A-F)** Quantitative analysis of mitophagy and autophagy markers in DRG tissues from *Fgf13*^fl/fl^ and SNS-Cre; *Fgf13*^fl/fl^ mice with or without AAV-shKIF1A injection. (A) p62, (B) Beclin1, (C) PINK1, (D) Parkin, (E) TOMM20, and (F) LC3-II/LC3-I ratio. Data are normalized to GAPDH and presented as fold of control. Data represent mean ± SEM; **p* < 0.05, ***p* < 0.01, ****p* < 0.001. Two-way ANOVA followed by Tukey’s multiple comparisons test was used in A-F.


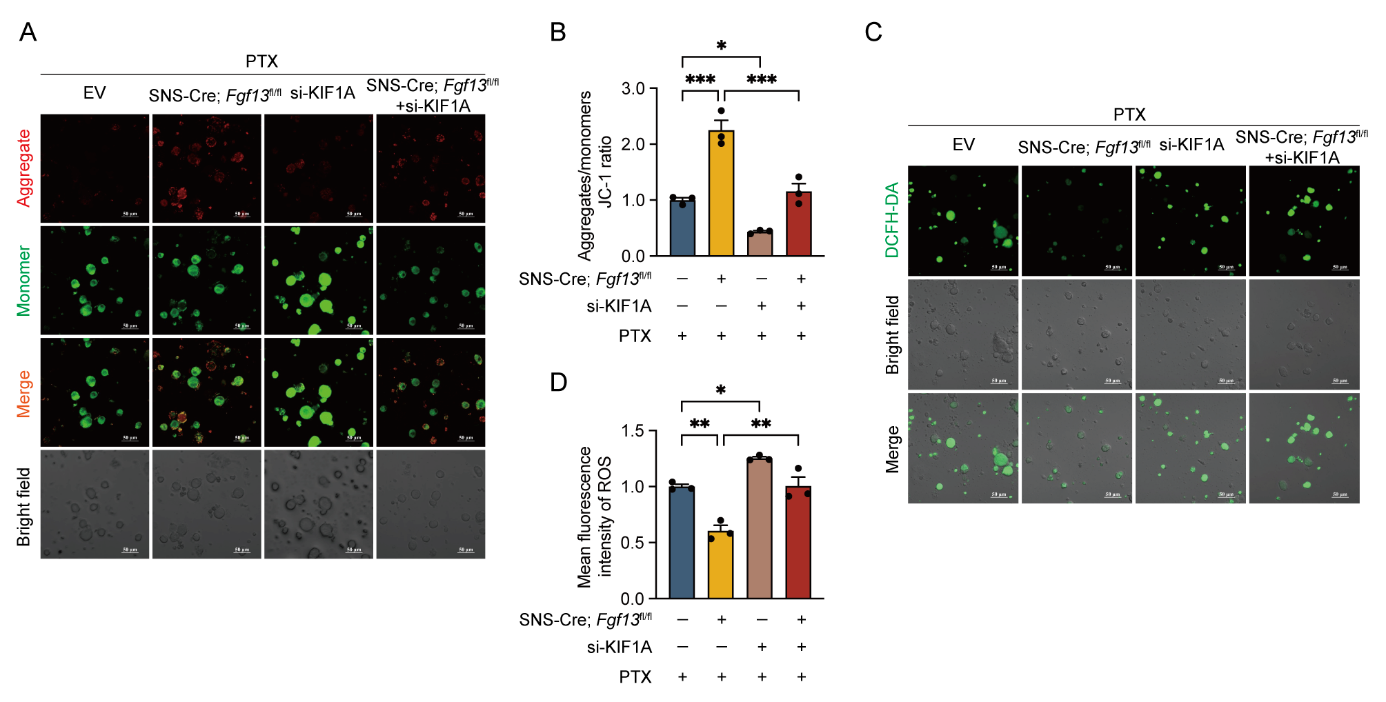


**Figure S10. KIF1A is essential for FGF13 deletion-enhanced mitophagy and attenuation of mitochondrial damage.**

**(A)** JC-1 staining measured ΔΨM in DRG neurons from *Fgf13*^fl/fl^ and SNS-Cre; *Fgf13*^fl/fl^ mice treated with si-NC or si-KIF1A under PTX stimulation. Scale bar = 50 μm. **(B)** The ratio of JC-1 aggregate to JC-1 monomer was compared in the bar graph (n = 3 per group). **(C)** Representative confocal images of DCFH-DA (green) staining in DRG neurons from *Fgf13*^fl/fl^ and SNS-Cre; *Fgf13*^fl/fl^ mice treated with si-NC or si-KIF1A under PTX stimulation. Scale bar = 50 μm. **(D)** Quantification of relative ROS fluorescence intensity in DRG neurons (n = 3 per group). Data presented as mean ± SEM. **p* < 0.05, ***p* < 0.01, ****p* < 0.001. Two-way ANOVA followed by Tukey’s multiple comparisons test was used in B, D.


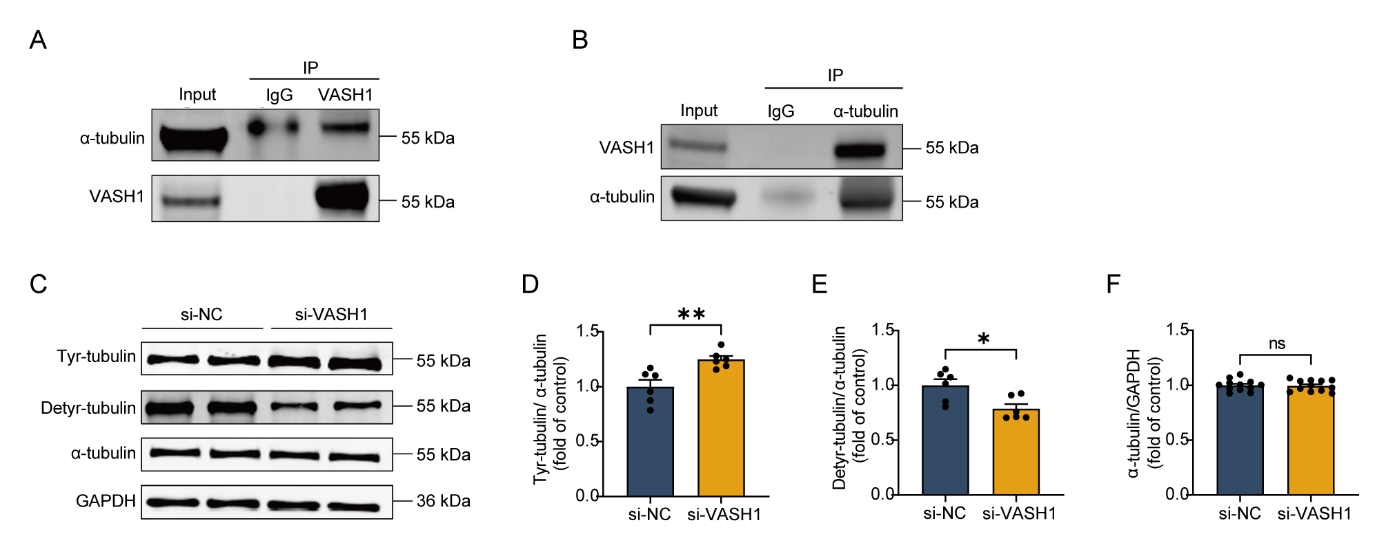


**Figure S11. VASH1 interacts with α-tubulin and promotes microtubule detyrosination.**

Interaction between VASH1 and α-tubulin in DRG neurons was examined by IP-western blotting assay. IP with VASH1 antibody **(A)** and IP with α-tubulin antibody **(B)**. IgG was used as control for IP. **(C)** Western blotting analysis revealed the expression levels of Tyr-tubulin, Detyr-tubulin, and α-tubulin in DRG neurons. **(D-F)** Quantitative analysis of Tyr-tubulin, Detyr-tubulin, and α-tubulin in the Western blotting experiments (n = 5 per group). Data presented as mean ± SEM. **p* < 0.05, ***p* < 0.01. ns, not significant. Two-tailed unpaired Student’s t-test was used in D-F. VASH1, vasohibin 1; Tyr -tubulin, Tyrosinated tubulin; Detyr-tubulin, Detyrosinated tubulin.


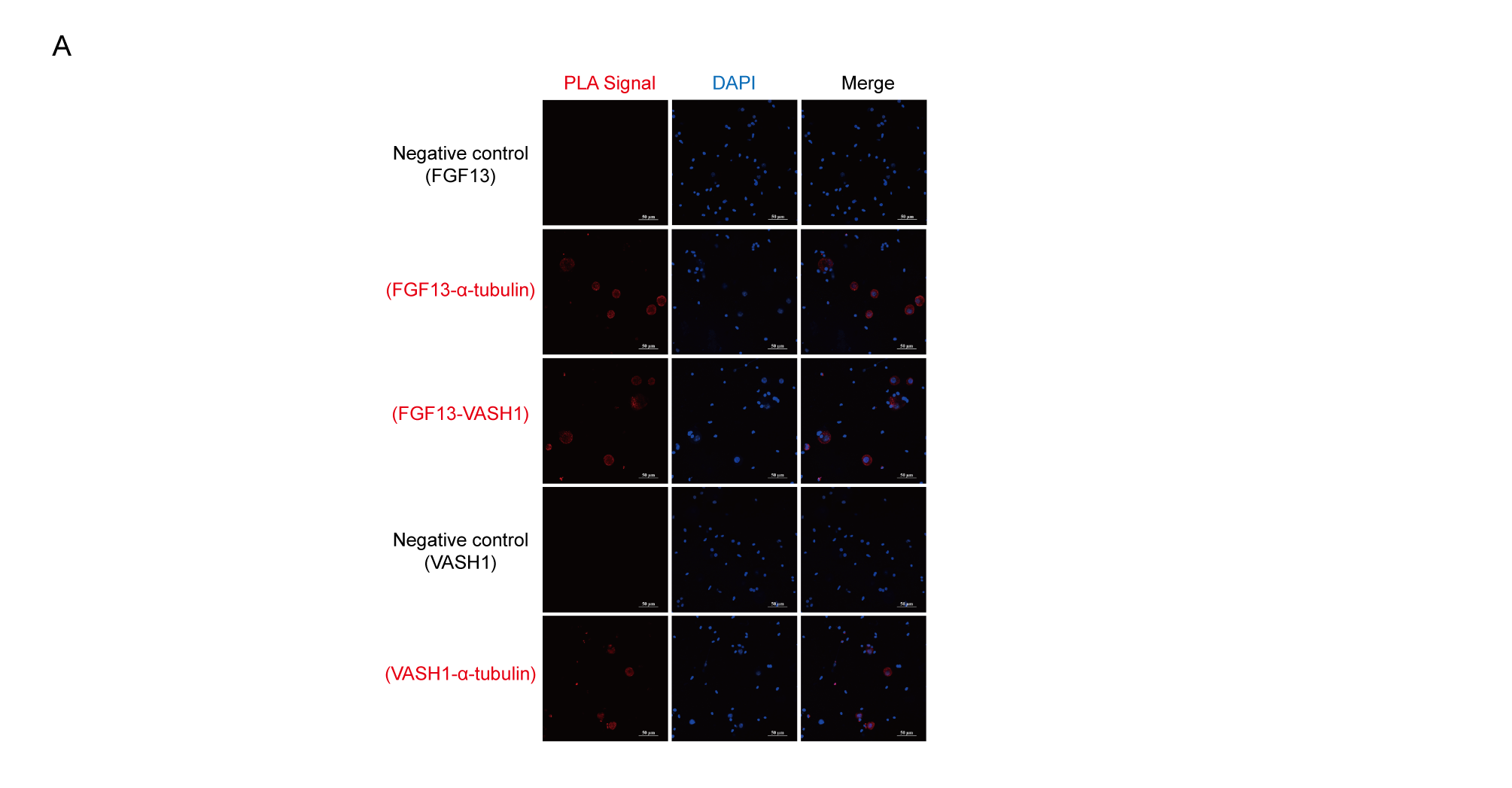


**Figure S12.** **FGF13, VASH1, and α-tubulin exhibit pairwise interactions.**

**(A)** Proximity ligation assay (PLA) in primary DRG neurons demonstrating close interactions between FGF13 and α-tubulin, FGF13 and VASH1, and VASH1 and α-tubulin. Scale bars, 50 μm.


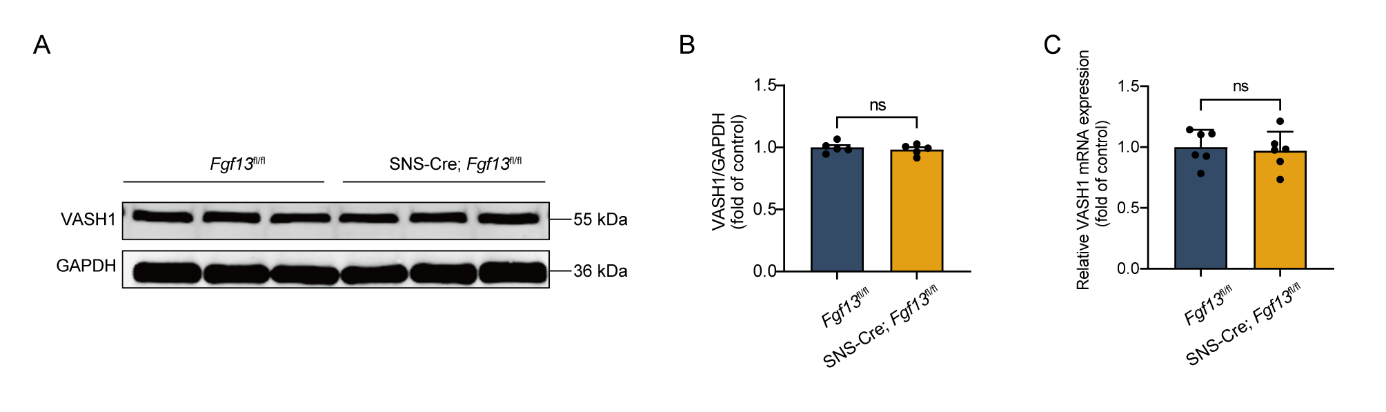


**Figure S13. The mRNA and protein levels of VASH1 did not change upon FGF13 knockout.**

**(A-B)** Western blotting and quantitative analysis of VASH1 in DRG tissues from *Fgf13*^fl/fl^ and SNS-Cre; *Fgf13*^fl/fl^ mice (n = 5 per group). **(C)** The VASH1 mRNA levels were analyzed by RT-qPCR (normalized to GAPDH) (n = 6 per group). Data presented as mean ± SEM. ns, not significant. Two-tailed unpaired Student’s t-test was used in B, C.


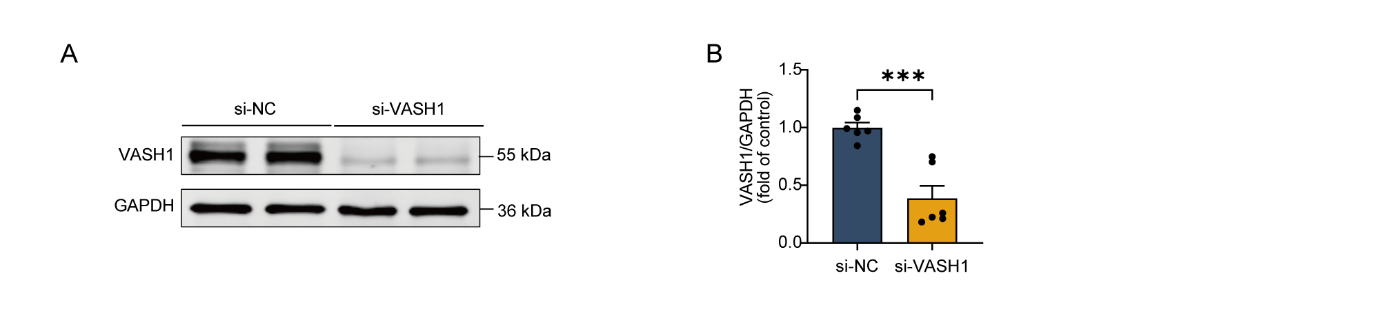


**Figure S14.** **Verification of the effects of** **VASH1 protein by a small interfering RNA for VASH1 in DRG** **neurons.**

**(A-B)** Western blotting and quantitative analysis of VASH1 expression were performed in DRG neurons transfected with si-VASH1 or si-NC (n = 5 per group). The protein level was standardized by GAPDH. Data are means ± SEM. Two-tailed unpaired Student’s t-test was used in B.


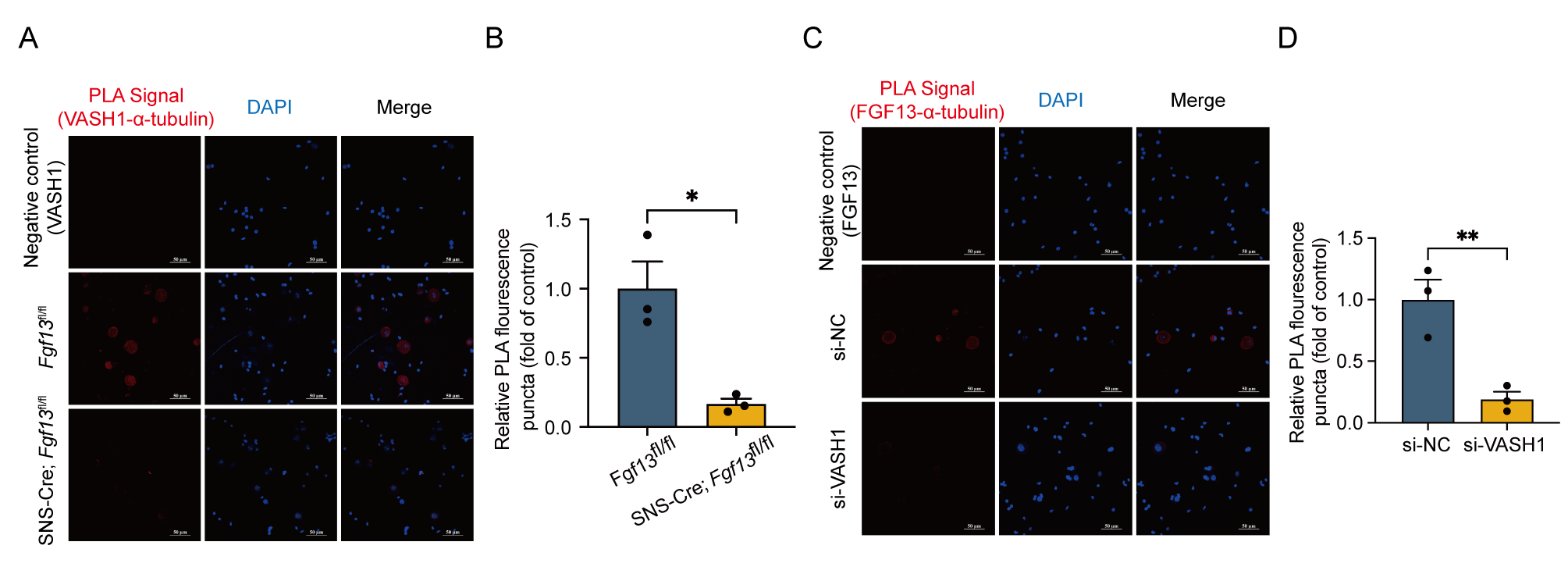


**Figure S15. FGF13, VASH1, and α-tubulin form a ternary complex via pairwise interactions.**

**(A-B)** PLA assay in primary DRG neurons from *Fgf13*^fl/fl^ and SNS-Cre; *Fgf13*^fl/fl^ mice showed the interaction between VASH1 and α-tubulin. Scale bars, 50 μm. **(C-D)** PLA assay in primary DRG neurons transfected with si-NC or si-VASH1 showed the interaction between FGF13 and α-tubulin. Scale bars, 50 μm. Data are means ± SEM. Two-tailed unpaired Student’s t-test was used in B, D.


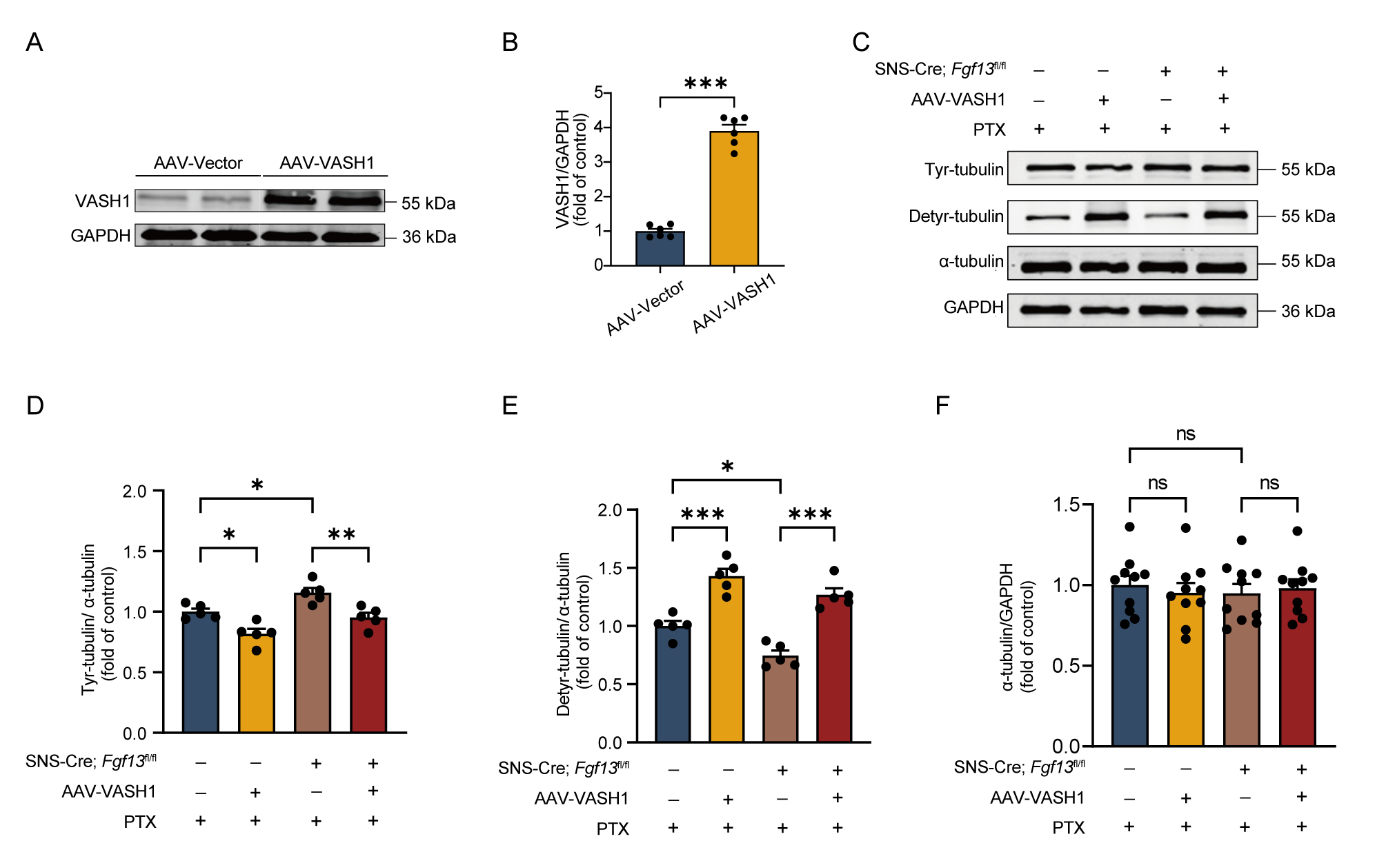


**Figure S16. DRG neuron-specific** **VASH1 overexpression in SNS-Cre; *Fgf13*^fl/fl^ mice increases microtubule detyrosination and reduces microtubule tyrosination.**

**(A-B)** Western blotting and quantitative analysis of VASH1 expression in DRG tissues from mice injected with AAV-hSyn-Vector or AAV-hSyn-VASH1 (n = 5 per group). **(C)** Western blotting analysis revealed the expression levels of Tyr-tubulin, Detyr-tubulin, and α-tubulin in DRG tissues. **(D-F)** Quantitative analysis of Tyr-tubulin, Detyr-tubulin, and α-tubulin in the Western blotting experiments (n = 5 per group). Data presented as mean ± SEM. **p* < 0.05, ***p* < 0.01, ****p* < 0.001. ns, not significant. Two-tailed unpaired Student’s t-test was used in B. Two-way ANOVA followed by Tukey’s multiple comparisons test was used in D, E, F.


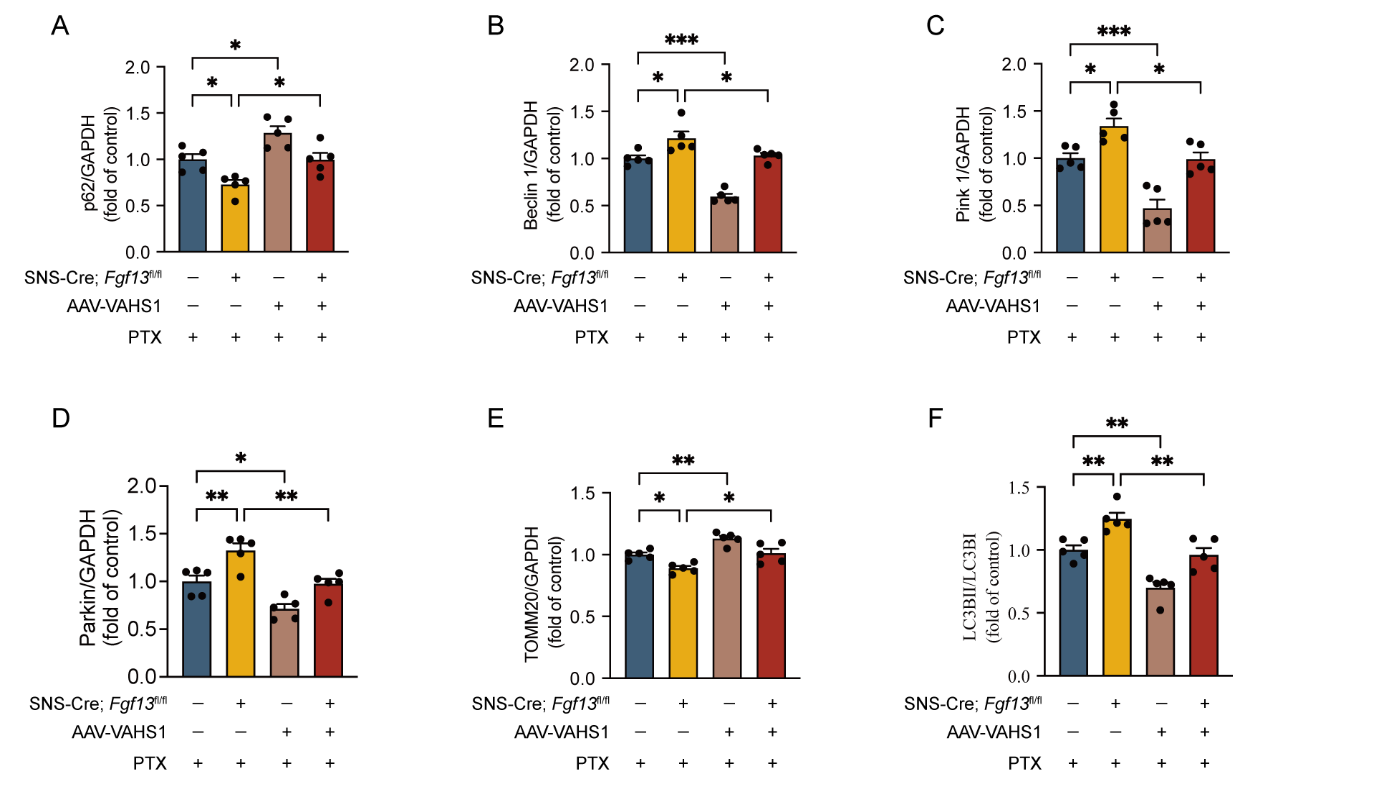


**Figure S17. FGF13 deficiency promotes mitophagy through VASH1-dependent mechanism in PIPNP.**

**(A-F)** Quantitative analysis of mitophagy and autophagy markers in DRG tissues from *Fgf13*^fl/fl^ and SNS-Cre; *Fgf13*^fl/fl^ mice with or without AAV-VASH1 injection. (A) p62, (B) Beclin1, (C) PINK1, (D) Parkin, (E) TOMM20, and (F) LC3-II/LC3-I ratio. Data are normalized to GAPDH and presented as fold of control. Data represent mean ± SEM; **p* < 0.05, ***p* < 0.01, ****p* < 0.001. Two-way ANOVA followed by Tukey’s multiple comparisons test was used in A-F.

**Supplementary Tables**

**Table S1. Genotyping primers and cycling conditions for** ***Fgf*13^fl/fl^ and SNS-Cre mice.**

| Genotype | Primer Sequence (5' to 3') |
| --- | --- |
| SNS-Cre-Forward | ATTTGCCTGCATTACCGGTC |
| SNS-Cre-Reverse | GCATCAACGTTTTCTTTTCGG |
| *Fgf13* ^fl/fl^-Forward | TAGTTCCATCTAACAGGGCTCATG |
| *Fgf13* ^fl/fl^-Reverse | AGACTTTGGTGGGAGCATCCTG |

The cycling conditions of *Fgf13*^fl/fl^ were: 94 °C for 3 min, and 95 °C for 15 s, 64.4 °C for 20 s, 72 °C for 1 min for 35 cycles, and 72 °C for 10 min. The cycling conditions of SNS-Cre were: 94 °C for 3 min, and 95 °C for 15 s, 55 °C for 20 s, 72 °C for 1 min for 35 cycles, and 72 °C for 10 min.

**Table S2. Primer sequences used for the RT-qPCR assays in this study.**

| Gene | Primer Sequence (5' to 3') |
| --- | --- |
| *Fgf13*-Forward | GTTAAGGAAGTCATATTCAGAGC |
| *Fgf13*-Reverse | CACCACCCGAAGACCCACAG |
| *Vash1*-Forward | ATGTGGAAGGCATGTGGCCAAG |
| *Vash1*-Reverse | CACCCGGATCTGGTACCCACT |
| *Gapdh*-Forward | TGTCAGCAATGCATCCTGCA |
| *Gapdh*-Reverse | CCGTTCAGCTCTGGGATGAC |
